# Delta Radiomic Features Predict Resection Margin Status and Overall Survival in Neoadjuvant-Treated Pancreatic Cancer Patients

**DOI:** 10.1245/s10434-023-14805-5

**Published:** 2023-12-27

**Authors:** Kai Wang, John D. Karalis, Ahmed Elamir, Alessandro Bifolco, Megan Wachsmann, Giovanni Capretti, Paola Spaggiari, Sebastian Enrico, Kishore Balasubramanian, Nafeesah Fatimah, Giada Pontecorvi, Martina Nebbia, Adam Yopp, Ravi Kaza, Ivan Pedrosa, Herbert Zeh, Patricio Polanco, Alessandro Zerbi, Jing Wang, Todd Aguilera, Matteo Ligorio

**Affiliations:** 1 Department of Radiation Oncology, University of Texas Southwestern Medical Center, Dallas, TX USA; 2 Department of Surgery, University of Texas Southwestern Medical Center, Dallas, TX USA; 3 Department of Pathology, Veterans Affairs North Texas Health Care System, Dallas, TX USA; 4 Pancreatic Surgery Unit, IRCCS Humanitas Research Hospital, Rozzano, Italy; 5 Department of Biomedical Sciences, Humanitas University, Pieve Emanuele, Italy; 6 Department of Pathology, IRCCS Humanitas Research Hospital, Rozzano, Italy; 7 Department of Radiology, University of Texas Southwestern Medical Center, Dallas, TX USA

## Abstract

**Background:**

Neoadjuvant therapy (NAT) emerged as the standard of care for patients with pancreatic ductal adenocarcinoma (PDAC) who undergo surgery; however, surgery is morbid, and tools to predict resection margin status (RMS) and prognosis in the preoperative setting are needed. Radiomic models, specifically delta radiomic features (DRFs), may provide insight into treatment dynamics to improve preoperative predictions.

**Methods:**

We retrospectively collected clinical, pathological, and surgical data (patients with resectable, borderline, locally advanced, and metastatic disease), and pre/post-NAT contrast-enhanced computed tomography (CT) scans from PDAC patients at the University of Texas Southwestern Medical Center (UTSW; discovery) and Humanitas Hospital (validation cohort). Gross tumor volume was contoured from CT scans, and 257 radiomics features were extracted. DRFs were calculated by direct subtraction of pre/post-NAT radiomic features. Cox proportional models and binary prediction models, including/excluding clinical variables, were constructed to predict overall survival (OS), disease-free survival (DFS), and RMS.

**Results:**

The discovery and validation cohorts comprised 58 and 31 patients, respectively. Both cohorts had similar clinical characteristics, apart from differences in NAT (FOLFIRINOX vs. gemcitabine/nab-paclitaxel; *p* < 0.05) and type of surgery resections (pancreatoduodenectomy, distal or total pancreatectomy; *p* < 0.05). The model that combined clinical variables (pre-NAT carbohydrate antigen (CA) 19-9, the change in CA19-9 after NAT (∆CA19-9), and resectability status) and DRFs outperformed the clinical feature-based models and other radiomics feature-based models in predicting OS (UTSW: 0.73; Humanitas: 0.66), DFS (UTSW: 0.75; Humanitas: 0.64), and RMS (UTSW 0.73; Humanitas: 0.69).

**Conclusions:**

Our externally validated, predictive/prognostic delta-radiomics models, which incorporate clinical variables, show promise in predicting the risk of predicting RMS in NAT-treated PDAC patients and their OS or DFS.

**Supplementary Information:**

The online version contains supplementary material available at 10.1245/s10434-023-14805-5.

Pancreatic ductal adenocarcinoma (PDAC) was responsible for 466,003 deaths worldwide in 2020 and is projected to be the second-leading cause of cancer-related deaths in the United States (US) before 2030.^1,2^ Treatment with neoadjuvant chemotherapy (NAT) has emerged as a standard of care for PDAC patients undergoing surgical resection. The goal of any resection in surgical oncology is to achieve, whenever possible, a negative (R0) resection margin. However, an analysis of the National Cancer Database showed that approximately one in six patients who undergo surgery receive an R1 resection, which might have an unfavorable impact on survival.^3,4^ Thus, additional tools are needed to identify patients who are most likely to receive an R0 resection after NAT, or whether alternative approaches (e.g., radiotherapy) should be applied prior to surgery to maximize our chances of an R0 resection in high-risk R1 positive-margin patients.

Radiographic assessment of pancreatic tumor response to NAT currently relies only on the measurement of tumor dimensions prior to and after receiving NAT. However, the change in the size of the primary tumor has been shown to be an unreliable marker of response to chemotherapy in PDACs.^5,6^ Furthermore, although the presence of abnormal soft tissue density in the peripancreatic fat prior to therapy predicts the presence of extra-pancreatic perineural invasion,^7^ the assessment of therapy response in such cases is even more challenging due to the NAT-induced fibrosis.^8^ Alternatively, an emerging radiological method based on the quantification of tumor features (i.e., radiomics features), such as enhancement, texture, shape, and heterogeneity, have shown promise in predicting the clinical outcomes of PDAC.^9^ Moreover, delta-radiomics features (DRFs), which capture quantitative changes between before, during, and after NAT in serial image representation, can be used to predict response and patient prognosis and have shown improved prognostic value compared with single timepoint radiomics features,^8,10–13,13–16^ Radiomic analysis also has the potential to gain insights into tumor biology, which is not currently available, and may complement future radiology review. However, to date, few studies have explored the prognostic value of DRFs, and to our knowledge there are no reports investigating whether DRFs can predict the likelihood of achieving an R0 resection margin. Therefore, we hypothesized that the development of a robust methodology to assess NAT response by applying DRFs could identify patients with favorable outcomes and predict the likelihood of an R0 resection prior to surgery. To this end, we developed, and externally validated, a novel delta radiomics-based model to predict overall survival (OS) and disease-free survival (DFS), and, most importantly, the likelihood of achieving an R0 resection in NAT-treated PDAC patients.

## Methods

This was a retrospective observational cohort study approved by the University of Texas Southwestern Medical Center (discovery cohort) and Humanitas Research Hospital (validation cohort) Institutional Review Boards. It was designed in accordance with the reporting guidelines proposed by the Image Biomarker Standardization Initiative (IBSI), and our methodology was self-assessed via the modified Radiomics Study Quality (mRQS) score^9,17–19^ and CheckList for EvaluAtion of Radiomics research (CLEAR).^20^ Inclusion criteria were (1) pathologically confirmed PDAC; (2) treated with NAT; and (3) contrast-enhanced computed tomography (CT) performed before and after NAT, while exclusion criteria were radiological data not available for analysis.

### Image Acquisition, Contouring, and Segmentation

Image acquisition characteristics are described in Table 1 of the electronic supplementary material (ESM). Contrast-enhanced CT scans were reviewed, and the gross tumor volume (GTV) was identified and contoured by an experienced radiation oncology team at the University of Texas Southwestern Medical Center (UTSW) [AE, TA], a high-volume center for pancreas radiation. Volumes were delineated on arterial phase scans and, if none were available, then on portal venous scan; a breakdown is shown in ESM Table 1. Patients were excluded whenever no contrast scans were available. The contrast phase was verified by subjective evaluation of enhancement in the aorta, liver parenchyma, and portal vein, and the presence of renal cortical/medullary enhancement. The GTV includes the tumor proper volume in addition to the heterogonous, infiltrating portion of the tumor extending out of the pancreas into adjacent soft tissue and mesenteric vessels, if applicable, as is common with standard radiation oncology volume delineation.^21–23^ CT contours were performed on the baseline CT scan prior to receipt of NAT (pre-NAT), as well as the CT scan performed following NAT (post-NAT). The post-treatment volume was determined by using pretreatment imaging tumor assessment as done in routine clinical radiation oncology practice. Image acquisition characteristics are described in ESM Table 1. Eclipse software (Varian Medical Systems, Palo Alto, CA, USA) was used for GTV contouring; contouring was performed blinded to margins and clinical outcomes in a retrospective fashion. Pre- and post-NAT volumes were not independently contoured. When the post-NAT tumor could not be clearly observed, the pretreatment volume was transferred from fused scans and modified using standard clinical judgment for a modified volume encompassing the key region where the tumor was known to be.

**Table 1:** Demographic, clinical, and treatment characteristics

	Discovery cohort	Validation cohort	*p*-Value
No. of patients	58	31	
Sex			0.07^b^
Male	35 (60.3)	12 (38.7)	
Female	23 (39.7)	19 (61.3)	
Age, years (IQR)	67.0 (60.0–73.0)	64.0 (60.3–69.8)	0.46^a^
BMI (IQR)	27.0 (23.1–29.3)	24.1 (21.8–26.2)	0.12^a^
History of smoking			0.12^b^
Yes	25 (43.1)	19 (61.3)	
No	33 (56.9)	12 (38.7)	
ECOG at diagnosis			NA
0	27 (46.6)	NA	
1	21 (36.2)	NA	
2	2 (3.5)	NA	
Unknown	8 (13.8)	NA	
Resectability status			0.07^b^
Resectable	32 (55.2)	13 (41.9)	
Borderline resectable	17 (29.3)	12 (38.7)	
Locally advanced	8 (13.8)	6 (19.4)	
Metastatic	1 (1.7)	0 (0.0)	
CA19-9			
Pre-neoadjuvant therapy (IQR)	290.5 (78.4–1038.5)	329.0 (18.3–962.2)	0.27^a^
Post-neoadjuvant therapy (IQR)	29.4 (15.0–96.3)	56.4 (11.8–256.9)	0.58^a^
Value decrease ≥50%	40 (69.0)	16 (51.6)	0.11^b^
Neoadjuvant chemotherapy regimen			**< 0.05**^**b**^
FOLFIRINOX	39 (67.2)	10 (3.2)	
Gemcitabine/*nab*-paclitaxel	16 (27.6)	12 (38.7)	
Gemcitabine	3 (5.2)	1 (3.2)	
Other	0 (0.0)	8 (25.8)	
Neoadjuvant radiation			0.24^b^
Yes	12 (20.7)	3 (9.7)	
No	46 (79.3)	28 (90.3)	
Surgery			**< 0.05**^**b**^
Pancreatoduodenectomy	41 (70.7)	16 (51.6)	
Distal pancreatectomy	16 (27.6)	7 (22.6)	
Total pancreatectomy	1 (1.7)	8 (25.8)	
Post-surgery follow-up, months (IQR)	18.9 (12.0–29.2)	16.4 (6.3–25.5)	0.42^a^

Data are expressed as *n* (%) unless otherwise specified

^a^Student’s *t*-test

^b^Fisher’s exact test

*IQR* interquartile range, *BMI* body mass index, *ECOG* Eastern Cooperative Oncology Group, *NA* not available, *CA19-9* carbohydrate antigen 19–9

### Clinical Data Collection

In addition to imaging data, we collected clinical information of the included patients. Most of the clinical data were preoperative patient characteristics, comprising sex, age, BMI, history of smoking, Eastern Cooperative Oncology Group (ECOG) performance status at diagnosis, clinical stage, NAT treatment method, CA19-9 before and after NAT, and primary tumor resectability status, i.e. resectable, borderline resectable, locally advanced, metastatic (see Table 1 for a summary of these data). Treatment effect score, T stage, N stage, number of excised nodes, number of positive nodes, lymphovascular invasion, and tumor margin status were also collected as postoperative features for constructing comparison survival prediction models. Of note, no postoperative clinical features were used to build the proposed DRF-based outcome prediction models.

### Image Preprocessing and Radiomic Feature Extraction

Image preprocessing and feature extraction was performed using MATLAB R2018b software (MathWorks, Natick, MA, USA). For each CT image, a total of 257 radiomics features were extracted using an open-source radiomics toolbox that satisfies the methodology and definitions of the IBSI.^17,24^ In the discovery cohort, four patients had biliary stents prior to NAT and 19 patients had biliary stents after NAT, while in the validation cohort, 6 patients had biliary stents prior to NAT and 9 patients had biliary stents after NAT. We used a threshold-based methodology to exclude volumes with Hounsfield Unit (HU) out of [− 200, 500], the high-intensity volume impacted by metal stents, as these can impact feature extraction from the target volumes.^25^ After excluding the region according to the mask of the stent, the no-stent image was used to extract the GTV. ESM Fig. 1 shows an example of images pre- and post-stent removal, and the GTV mask. The extracted radiomics features included nine intensity features (minimum, maximum, mean, standard deviation, sum, median, skewness, kurtosis, and variance), eight geometry features (GTV volume, major diameter of GTV, minor diameter of GTV, eccentricity, elongation, orientation, bounding box volume, and perimeter of the GTV on the slice that has the biggest tumor area), and 240 texture features (9 features from the Gray-Level Co-Occurrence Matrix [GLCM], 13 features from the Gray-Level Run-Length Matrix [GLRLM], 13 features from the Gray-Level Size Zone Matrix [GLSZM] and 5 features from the Neighborhood Gray-Tone Difference Matrix [NGTDM] collected under six different intensity levels). The GTVs were isotopically resampled to 1 × 1 × 1 mm^3^ voxels for feature extraction (ESM Table 2). Three-dimensional (3D) DRFs were calculated by direct subtraction of pre-NAT and post-NAT radiomic features.^26–28^ Additionally, we combined the pre-NAT radiomics features and DRFs equally between the two scans as the radiomics feature input, which is a vector of 514 features per patient. We termed the concatenated baseline and delta-radiomics features as ‘BL-DRF’.^9,11^

**Fig. 1:**
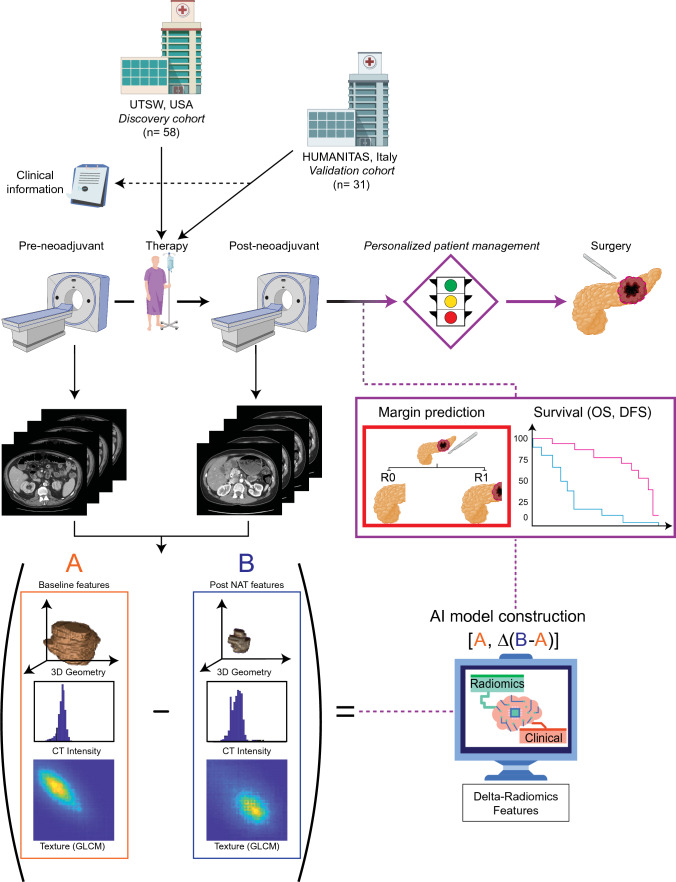
Study workflow. *UTSW* University of Texas Southwestern Medical Center, *OS* overall survival, *DFS* disease-free survival, *NAT* neoadjuvant therapy, *3D* three-dimensional, *CT* computed tomography, *GLCM* Gray-Level Co-Occurrence Matrix

### Feature Selection and Model Construction

Three sets of models were fitted using the discovery cohort: (1) a model consisting of demographic and preoperative clinical variables (PC); (2) the BL-DRF model; and (3) a combined BL-DRF and preoperative clinical variable model (BL-DRF-PC). A multivariable Cox proportional hazards model was constructed to identify the association of each model’s feature with OS and DFS. We also constructed a binary prediction model for R0 margin prediction. The workflow of feature selection and model construction is depicted in ESM Fig. 2. In addition, we compared our models with some baseline models, which are widely used in the field of radiomics, some of which have been used in pancreas cancer-related studies,^11,29–31^ and analyzed the final model (BL-DRF-PC) in patients with a different resectability status (i.e., resectable, borderline resectable, and locally advanced). For OS and DFS prediction (ESM Table 4), we compared our results with preoperative and postoperative clinical models. We then built a series of radiomic-based models with the objective of outperforming clinically based models. Specifically, we assessed five distinct radiomics models:

- A pre-NAT radiomics model, constructed using baseline radiomic features extracted from pre-NAT CT images.
- A post-NAT radiomics model, derived from baseline radiomic features sourced from post-NAT CT images.
- A DRF model, which incorporated the differences between pre-NAT and post-NAT features in CT images.
- Pre-NAT radiomic features plus DRFs (BL-DRF), which incorporated pre-NAT radiomic features with DRFs.
- Relative delta-radiomics feature model (BL-Relative-DRF), which incorporates DRFs as a relative change of baseline radiomics features (${{\text{DRF}}}_{{\text{relative}}}=\frac{{{\text{Feature}}}_{{\text{post}}}-{{\text{Feature}}}_{{\text{pre}}}}{{{\text{Feature}}}_{{\text{pre}}}}$).

**Fig. 2:**
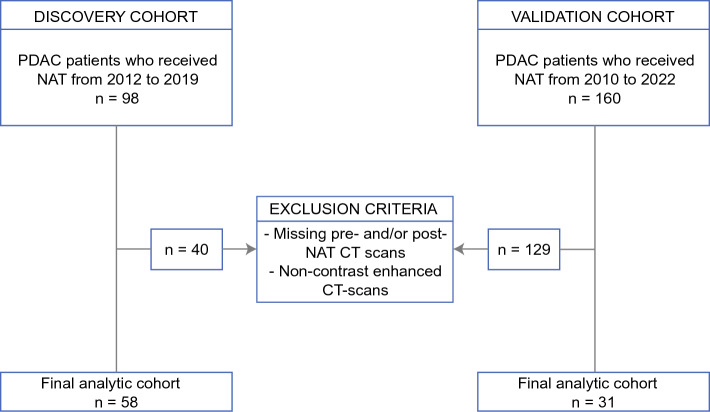
Study cohort eligibility criteria. *PDAC* pancreatic ductal adenocarcinoma, *NAT* neoadjuvant therapy, *CT* computed tomography

For resection margin status prediction, in addition to different DRFs, we compared our method with two baseline models widely used in radiomics studies: (1) a logistic regression model; and (2) a support vector machine model.^32–35^ The least absolute shrinkage and selection operator (LASSO) method is used for feature selection for these two models. Models constructed with a preoperative clinical feature only, DRF only, and combined clinical and delta-radiomics were evaluated for comparison.

We first performed a 100 times fivefold cross-validation on the discovery cohort for preoperative clinical features, each model design and BL-DRF preselection separately. The partition of the training and validation sets was random each time. Univariate Cox regression models were built for OS and DFS prediction, and univariate logistic regression models were built for R0 margin prediction. The average C-index of univariate OS and DFS prediction models, and area under the curve (AUC) of univariate R0 margin prediction on validation data were recorded and the order of features were sorted based on the validation performance. To avoid features with a low-predictive ability, features that had an AUC or C-index of < 0.5 were removed from the feature set corresponding to the prediction task. To reduce redundancy in the feature sets for different prediction targets, we performed a Spearman rank correlation analysis for all features. Any feature with an absolute correlation coefficient > 0.9 to any of its previous features was removed from the feature set for the corresponding prediction target.

For OS and DFS prediction, we performed a multivariate Cox proportional hazard regression with step-forward feature selection strategy to further select predictive features and construct the survival models. Outcomes analysis was performed at the patient level, given 3D radiomic features and BL-DRF combination linking the pre- and post-NAT scans into a feature. The clinical models and radiomic models were built separately. The C-index was the criteria for step-forward feature selection. Five was set as the maximum number of selected features. An additional 100 times fivefold cross-validation was conducted to mitigate the impact of random patient partition, and the same partition was used in each of the fivefold cross-validations to construct preoperative clinical and BL-DRF models. The risk scores of the training and validation samples in each of the fivefold cross-validations were recorded. The average validation C-indices were used as the final survival prediction performance for the OS and DFS models in the discovery cohort. The risk scores of the preoperative clinical survival models and BL-DRF models on training data were then averaged as the risk score from the combined models for OS and DFS prediction. The average risk scores from the preoperative clinical model, BL-DRF model, and BL-DRF-PC model were recorded and were used to differentiate high- and low-risk patient groups separately; the thresholds were set as the median risk scores. We also used these risk scores as the only feature to predict binary patient outcomes, including patient death and cancer recurrence, at 1, 2, and 3 years of follow-up. For each time point, patients who had shorter follow-up than the evaluated time interval and did not have a corresponding event (death or disease recurrence) were censored.

For margin status prediction, we used a multi-classifier, multi-objective, multi-modality (mCOM) binary outcome prediction model to predict R0 margin, which sets sensitivity and specificity as the objectives simultaneously; adopts support vector machine (SVM), logistic regression, and discriminant analysis together to construct the model; and fuses the output probabilities from single-modality models to offer the final prediction. The mCOM model is optimized via an iterative multi-objective immune algorithm (IMIA). The optimization process includes further feature selection, classifier parameter selection, and weighting factor selection. For model details regarding the mCOM model and the IMIA, please refer to the ESM included with this article, as well as our previous work.^36,37^

### Model External Validation

For OS and DFS prediction, we retrained all Cox proportional hazards models using the final selected feature sets with the whole discovery cohort data to generate one set of models and evaluated their performance with the validation cohort. Risk scores of the validation data were recorded for measuring the C-indices and differentiating high- and low-risk patient groups. For R0 margin prediction, as the validation data are always necessary for the mCOM model, we did not retrain the model, but ensembled the output probabilities of the generated models while repeating fivefold cross-validation. Model evaluation metrics comprising sensitivity, specificity, accuracy, and AUC were measured based on the ensembled output probability values of the validation data.

### Margin Resection Status

Margin status was defined identically at both centers following the College of American Pathologists (CAP) criteria. Specifically, R1 resection is defined as a tumor that is present within 1 mm from the resection margins in the final pathology report. Our final mCOM model was also tested in patients with a different resectability status (i.e., resectable, borderline resectable, and locally advanced) to compare model performance in each patient subgroup.

### Statistical Analysis

Statistical analyses were performed with Python software using the scikit-learn library and lifelines library. The Fisher’s exact test and Student’s *t*-test were used to compare categorical and continuous variables, respectively, and a paired *t*-test was used to compare the C-indices from different survival prediction models for survival prediction tasks. Survival outcomes of differentiated high- and low-risk patient groups using different survival prediction models were compared via the log-rank test. Performance of the binary prediction models was evaluated by calculating the AUC and Delong’s test. For all statistical analysis, a *p*-value of ≤ 0.05 was considered significant.

## Results

The workflow to identify, test, and validate the DRF as a predictive and prognostic biomarker for patients with pancreatic cancer is presented in Fig. 1. A discovery and validation cohort were curated, CT scans were identified, and clinical outcomes were determined for model development. The modified radiomics quality score (mRQS) for this study was 16/36 (ESM Table 7) and was much higher than the 7.5/36 that was recently observed in recently published work.^9,11^ In addition, we satisfied 47 of the 58 items in the Checklist for Evaluation of Radiomics Research (CLEAR) checklist (ESM Table 8), where the unaccomplished items included two non-applicable and nine unsatisfied items.

### Patient Cohorts

Patients with pathologically confirmed PDAC who were treated with NAT were screened for inclusion (Fig. 2). Patient demographic, clinical, and pathologic characteristics are summarized in Table 1. Overall, 58 patients met the criteria for inclusion in the discovery cohort (i.e., UTSW, USA) (Fig. 1). The median age at diagnosis was 67 years (interquartile range [IQR] 60–73 years), 60% were males, and 83% of patients had an ECOG performance status of 0 or 1. A subset of patients (*n* = 43%) had borderline or locally advanced tumors at diagnosis. 12/58 (21%) patients received neoadjuvant radiation in addition to NAT, and FOLFIRINOX and gemcitabine/nab-paclitaxel were administered in 67% and 28% of patients, respectively. Borderline and locally advanced pancreas cancer determination was made by clinical assessment by the multidisciplinary team, but the vessel involvement was verified at the time of tumor volume delineation.

The validation patient cohort came from an independent center (HUMANITAS, Italy) with similar practice patterns (Fig. 1). Only 31 patients from Humanitas met the inclusion/exclusion criteria reported above. The discovery and validation cohorts had similar demographic and clinical characteristics, except for increased receipt of FOLFIRINOX in the discovery cohort (67% vs. 3%; *p* < 0.05) and an increased number of patients who underwent pancreatoduodenectomy in the discovery cohort (71% vs. 52%; *p* < 0.05).

### Delta Radiomic Feature Model Development

We constructed a multivariate Cox proportional hazards model to identify the association of DRFs with OS. We generated multiple models based on radiomic features extracted from (1) pre-NAT CT scans; (2) DRFs; and (3) BL-DRF (please also see the ‘Feature Selection and Model Construction’ paragraph in the Methods section). Among these models, we found that the BL-DRF model had the strongest association with OS (C-index 0.73, 95% confidence interval [CI] 0.72–0.74) (ESM Table 4).

**Fig. 3:**
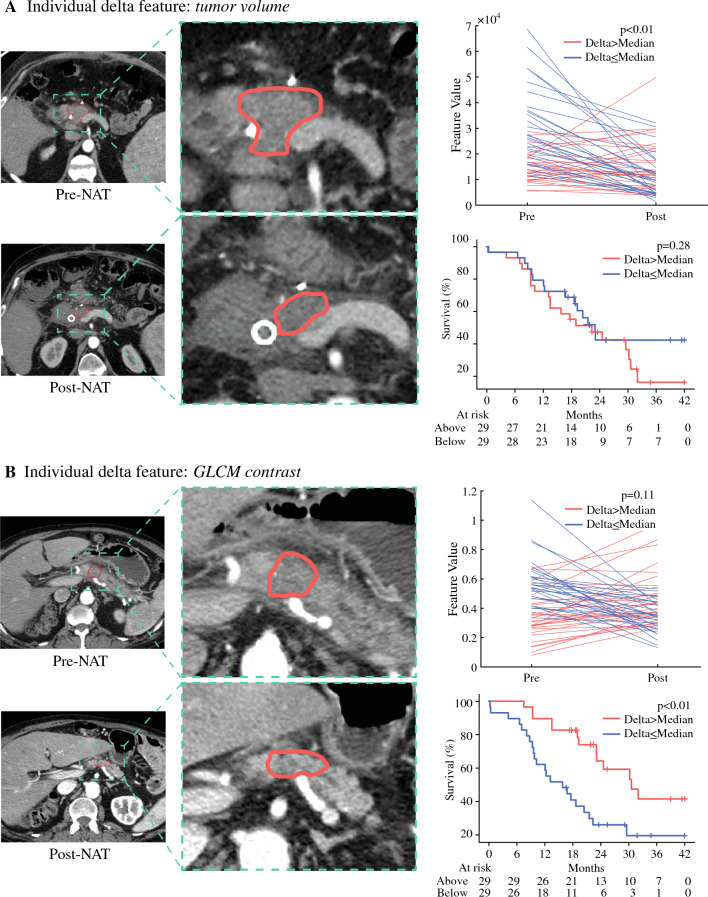
Examples of contoured pre- and post-NAT tumors are depicted to show the clinical relevance of different radiomic features. **A** Change in tumor volume that was not associated with overall survival (*p* = 0.28). **B** GLCM feature that was significantly associated with overall survival (*p* < 0.01). *NAT* neoadjuvant therapy, *GLCM* Gray-Level Co-Occurrence Matrix

We then reviewed the individual features selected by the model and examined their association with OS. Notably, the change in tumor volume after NAT, a metric readily measurable by standard radiologist review, was not associated with OS (Fig. 3a). We then reviewed the association of DRFs with OS, and found that the GLCM-derived ‘contrast’ was associated with OS (Fig. 3b).

### Integration of Delta Radiomic and Clinical Features

**Fig. 4:**
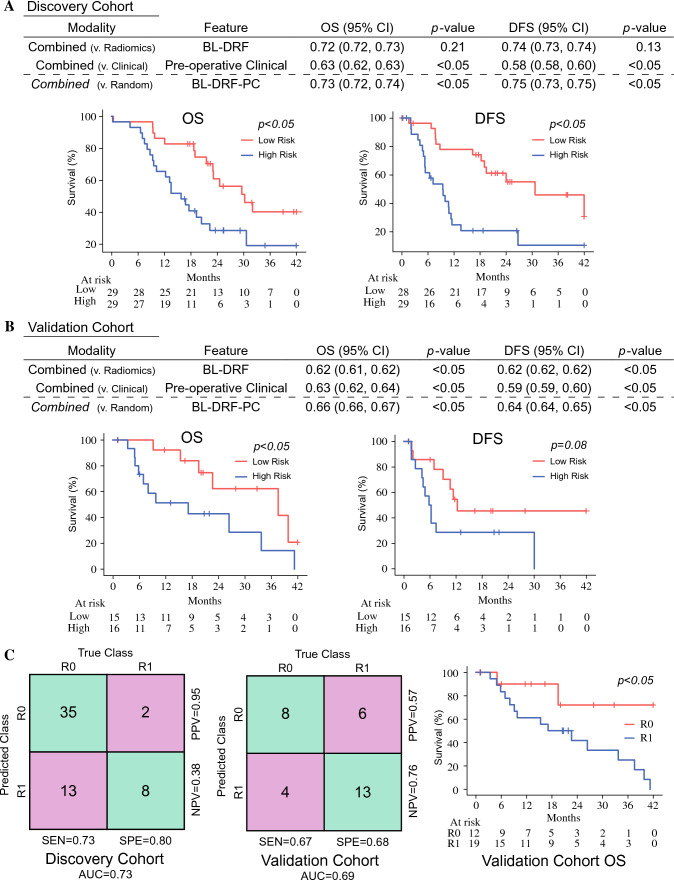
PDAC patient survival and margin status prediction. OS and DFS prediction performance of a concatenated BL-DRF model, preoperative clinical feature model, and a combined BL-DRF and preoperative clinical variable (BL-DRF-PC) model in the **A** discovery cohort and **B** validation cohort. **c** BL-DRF-PC prediction of surgical margin status in the discovery and validation cohorts. Kaplan–Meier curve showing the difference in survival based on margin status in the validation cohort. *PDAC* pancreatic ductal adenocarcinoma, *OS* overall survival, *DFS* disease-free survival, *BL-DRF* baseline and delta-radiomics feature, *CI* confidence interval

We constructed three sets of models (i.e., clinical, radiomics, and combined) and determined their association with survival in the discovery and validation cohorts (see ESM Table 4; the ‘Feature Selection and Model Construction’ paragraph in the Methods section; and section C [‘Workflow of Feature Selection and Model Construction’] in the ESM). The first model was solely based on preoperatively available clinical factors (i.e., preoperative clinical model, or PC). The factors selected for inclusion in the PC model were pre-NAT CA19-9, change in CA19-9 after NAT receipt (∆CA19-9), and primary tumor resectability status. The C-indices for this model for OS and DFS prediction in the discovery cohort were 0.63 (95% CI 0.62–0.63) and 0.58 (95% CI 0.58–0.63), respectively (ESM Table 4). We also built a postoperative clinical model (OS C-index: 0.719; DFS C-index: 0.662) and a model based on BL-DRFs (ESM Table 4), finding that the C-indices of the BL-DRF model for OS and DFS prediction were higher than the preoperative clinical model for OS (C-index: 0.72, 95% CI 0.72–0.73) and DFS (C-index 0.74, 95% CI 0.73–0.74). We then sought to determine if a model that combined BL-DRF and preoperative clinical factors (BL-DRF-PC) had a stronger prognostic value than the BL-DRF model alone. We found that our BL-DRF-PC model was most predictive of OS (C-index 0.73, 95% CI 0.72–0.74) and DFS (C-index 0.75, 95% CI 0.73–0.75) (Fig. 4a), even in comparison with the BL-Relative-DRF-PC model (OS C-index: 0.711; DFS C-index: 0.703), which included the relative DFR change (Table 4). We then evaluated our models with the validation cohort and found that the BL-DRF-PC model was predictive of OS (C-index 0.66, 95% CI 0.66–0.67) and DFS (C-index 0.64, 95% CI 0.64–0.65) (Fig. 4b). In addition, we compared the BL-DRF-PC model in patients with a different resectability status (Table 1) in both patient cohorts (ESM Fig. 3 and ESM Fig. 4).

### Margin Status Prediction

In the discovery cohort, the AUC for margin status prediction was 0.73, with a sensitivity of 73% and specificity of 80%. The average number of selected features for the mCOM model was five. Notably, the positive predictive value for achievement of an R0 margin was 95%. Similar results were observed in the validation cohort; the AUC was 0.69, with a sensitivity of 67% and a specificity of 68%, but we observed a negative predictive value for achievement of an R1 margin of 76%. To highlight the clinical value of these predictions, we plotted the Kaplan–Meier curves and observed an increase in survival in R0-resected patients compared with patients who received an R1 resection (Fig. 4c). We then compared our tumor margin status prediction models (mCOM) with other baseline models and logistic regression and support vector machine models (ESM Table 4 and ESM Table 5) and found that our mCOM outperformed the other two models in both the discovery cohort (mCOM, and logistic regression and support vector machine models: 0.73, 0.66, and 0.68) and the validation cohort (0.69, 0.61, and 0.65). In addition, we compared the mCOM model in patients with a different resectability status (ESM Table 9).

## Discussion

In this study, we built and externally validated a set of predictive/prognostic models that combined delta radiomic features with traditional clinical parameters (primary tumor resectability status, baseline, and change in CA19-9), to effectively assess the resection margin (R0 vs. R1 status) and survival (both OS and DFS) in NAT-treated PDAC patients prior to surgery. We were intrigued by the fact that DRFs, which capture the radiomic changes in the pre- versus post-NAT CT scans, improved our model prediction over models that were built with only pre- and/or post-NAT radiomic features (ESM Table 4 and ESM Table 5). The overall performance of our proposed combined model (BL-DRF-PC) was then compared with other models that were built using only pre-/postoperative clinical data and pre-/post- or delta-radiomic features (as specified above) and found that our combined clinical radiomic-based model (BL-DRF-PC) outperformed all other preoperative models (Fig. 4, ESM Table 4, and ESM Table 5). Overall, we believe that if prospectively validated by future clinical trials, our BL-DRF-PC model could complement the standard radiologic report and could have the potential to help guide clinical and surgical patient management.

Currently, the radiographic response and measurement of CA19-9 after receipt of NAT (Table 1) are the key parameters used to guide patient selection for surgery with curative intent. However, in our dataset, similar to previously published studies, a reduction in tumor size does not correlate with OS (Fig. 3a) and up to 22% of PDAC patients have tumors that do not secrete CA19-9.^5,6,38^ Thus, novel predictive methods are needed to estimate patients’ margin status, with the goal of having novel clinical tools that can guide the utilization of alternative strategies (e.g., radiotherapy) in conjunction with NAT if positive margins are anticipated.

Surgery remains the mainstay of treatment for localized PDAC. However, the morbidity of pancreatic surgery is high, and identifying which patients are most likely to benefit from a resection is an urgent need and challenge in the pancreatic field.^39^ Moreover, there is not a uniform consensus as to whether R1 positive margin status impacts long-term survival, but it is widely accepted that an R0 resection is always preferable. This exposes R1-resected patients to the risks and attendant morbidity of a large operation without the potential survival benefit.^3,40,41^

Our report represents one of the earlier studies^42^ to use a delta radiomics model to predict the ability to achieve an R0 resection margin in PDAC-resected patients, and although future prospective validation is needed, our work is one of the first externally validated studies that shows how preoperative radiomics models can provide clinicians with a powerful tool to improve patient selection for identifying the best surgical candidates. Furthermore, the high throughput and dynamic nature of delta radiomics allows for the discovery of predictive and prognostic features that are objective and quantitative, which are otherwise not captured in standard radiologic assessment. For example, Khalvati et al. identified radiomic features that had greater utility for the prognostication of PDAC patients who underwent a surgical resection when compared with conventional imaging features.^43^ Similarly, other studies have proposed prognostic models in multiple tumor types based on pretreatment radiomic features;^12,44–47^ however, the majority of these models extracted radiomics features from a single point in time (i.e., not a delta approach). A unique attribute of our study is the incorporation of pre- and post-NAT radiomic features to capture DRFs. In addition, the advantage of our delta radiomics model is, as mentioned, its dynamic nature, allowing to measure changes occurring in the tumor during NAT with the potential to predict/assess the impact of a specific treatment in real time (i.e., predictive biomarker) and gain biological insight in tumor biology. Thus, in our opinion, delta radiomics may improve our ability to more precisely distinguish treatment-responsive tumors from non-treatment-responsive tumors, with the following adjustments in patient management.

The discrepancy in model performance between the discovery and validation cohorts observed in our study may be explained by the slightly different NAT regimen used in the two centers (Table 1). For example, in the UTSW discovery cohort, a higher proportion of patients received FOLFIRINOX, whereas patients in the Humanitas validation cohort received a higher proportion of gemcitabine/*nab*-paclitaxel. Additionally, tumor location varied between cohorts, which resulted in different surgical approaches. In the UTSW cohort, a higher proportion of patients underwent pancreaticoduodenectomy, whereas in the Humanitas cohort, the majority of patients underwent total pancreatectomy (Table 1).

Our study has several limitations, mainly due to the retrospective nature of our analysis. As listed above, differences in the neoadjuvant regimens, use of radiation therapy, and the surgical technique can account for differences in DRFs across cohorts and have an impact on model performance (e.g., AUC, sensitivity, and specificity). While both institutions used the same margin assessment definitions, it is possible that a difference in histopathological technique could be found. Yet, we believe that these differences, which can be considered as limitations of our study, may actually highlight the large applicability of our BL-DRF-PC model across different centers, given the retaining significant predictive value in our external validation cohort (Fig. 4c). Another limitation of our study is the small sample size of our discovery and validation cohorts, even though it seems that the signal picked by our models is robust enough to meet the quality assessment criteria of IBSI and mRQS and to discern differences in radiomic features between two independent centers. Future larger-scale studies, ideally performed in a prospective fashion, would be needed to confirm/disprove our findings and evaluate the model functionality on different patient subgroups. Of note, our study paves the way for future investigations into the molecular mechanisms of radiomics-based PDAC chemoresponse, as well as for work that integrates radiomics with genetic, genomics, and transcriptomics data.

## Conclusion

In this study, we showed that our externally validated, predictive/prognostic models, combining DRFs and patient clinical characteristics, show promising results in estimating the risk of positive resection margin in NAT-treated PDAC patients, as well as their OS and DFS. If prospectively validated, especially in larger international consortia, our model has the potential to become a useful decision-making tool, providing an objective quantifiable tumor response assessment in conjunction with standard clinical, biochemical, and radiographic considerations.

### Supplementary Information

Below is the link to the electronic supplementary material.

Supplementary file1 (DOCX 1015 kb)
