# Supplementary material for: Delta Radiomic Features Predict Resection Margin Status and Overall Survival in Neoadjuvant-Treated Pancreatic Cancer Patients
Source: Ann Surg Oncol. 2023 Dec 27;31(4):2608–20. doi: 10.1245/s10434-023-14805-5 (PMC10908610; doi:10.1245/s10434-023-14805-5)
Supplement: Supplementary file 1 — Supplementary file1 (DOCX 1015 kb) [file 10434_2023_14805_MOESM1_ESM.docx]

**Delta radiomic features predict resection margin status and overall survival in neoadjuvant-treated pancreatic cancer patients**

**SUPPLEMENTARY MATERIALS**

1. **Details about imaging protocol**

In this study, the discovery cohort included data from 58 patients treated at UT Southwestern Medical Center (UTSW), Dallas, USA, the validation cohort included data from 31 patients treated at Humanitas Research Hospital (Humanitas), Rozzano, Italia. Most the enrolled patients have two contrast-enhanced CT (CECT) scans performed before and after neoadjuvant therapy (NAT). We summarized their pre- and post-NAT CECT imaging protocols in Table S1 below. Arterial scans were primarily used but when not available portal venous were accepted. Verification of the scan phase was made by the radiation oncology team analyzing the images and scan breakdowns are listed in Supplementary Table S1.

**Supplementary Table 1**: Imaging protocols of pre- and post-neoadjuvant therapy contrast-enhanced CT.

| **Discovery Cohort** (N=58×2) | | **Validation Cohort** (N=31×2) | |
| --- | --- | --- | --- |
| Term | Number | Term | Number |
| **Manufacturer** |  | **Manufacturer** |  |
| GE MEDICAL SYSTEMS | 59 (50.9%) | GE MEDICAL SYSTEMS | 21 (33.8%) |
| TOSHIBA | 25 (21.6%) | TOSHIBA | 11 (17.7%) |
| Philips | 17 (14.7%) | Philips | 26 (41.9%) |
| SIEMENS | 15 (12.9%) | SIEMENS | 4 (6.45%) |
| **Pixel Spacing (mm)** | 0.75 (IQR: 0.70-0.82) | **Pixel Spacing (mm)** | 0.77 (IQR: 0.71-0.80) |
| **Slice Thickness (mm)** | 3.75 (IQR: 3.00-5.00) | **Slice Thickness (mm)** | 2.50 (IQR: 2.50-5.00) |
| **Focal Spot (mm)** |  | **Focal Spot (mm)** |  |
| 1.2 | 74 (63.8%) | 1.2 | 23 (37.1%) |
| Other | 24 (20.7%) | Other | 11 (17.7%) |
| Unknow | 18 (15.5%) | Unknow | 28 (45.2%) |
| **Tube Voltage (kVp)** |  | **Tube Voltage (kVp)** |  |
| 120 | 106 (91.38%) | 120 | 54 (87.1%) |
| Other | 10 (8.62%) | Other | 8 (12.9%) |
| **Tube Current (mA)**  **Scan Phase (**Art/Ven/other) | 222.5 (IQR: 146.5-323.5)  78/32/6 | **Tube Current (mA)**  **Scan Phase (**Art/Ven/other) | 254 (IQR: 168-308)  56/6/0 |

1. **Details about feature extraction**

For each image, a total of 257 radiomics features were extracted using an open-source radiomics toolbox which satisfies the methodology and definitions of the Image Biomarker Standardization Initiative.^1,2^ The image values are in raw Hounsfield (HU) Unit format. All the images were resampled to voxel size of 1$\times$1$\times$1 mm^3^ before feature extraction. The extracted radiomics features included nine intensity features, eight geometry features, and 240 texture features. Pre- and post-treatment radiomics features were extracted from the delineated 3D volume of GTVs. If there was a stent present, then voxels with high Z metal were systematically removed (Supplementary Figure 1).


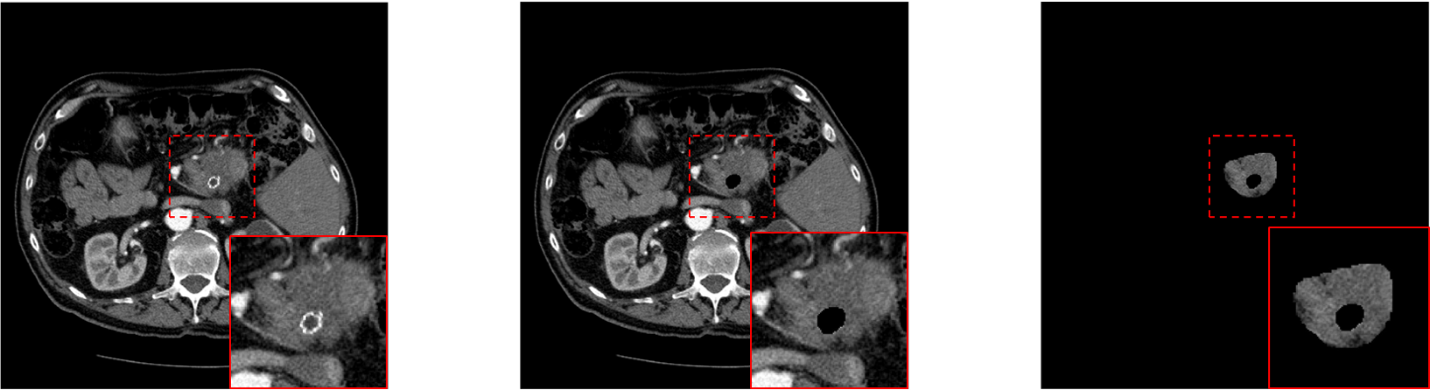


**Supplementary Figure 1.** Workflow for stent artifact renewal. The stent was removed by filtering out [-100, 300] HU with initial image on left, and artifact removal in the middle. The final volume contained GTV mask volume with the post artifact removed as shown in the image on the right.

Intensity features included minimum, maximum, mean, standard deviation, sum, median, skewness, kurtosis, and variance. Geometry features included GTV volume, major diameter of GTV, minor diameter of GTV, eccentricity, elongation, orientation, bounding box volume, and perimeter of the GTV on the slice which has the biggest tumor area. Texture features describe the intertumoral heterogeneity through quantitative measurement of the spatial distribution of the intensities within the ROI (GTV in our study). Following Vallieres et al.^1^, we computed nine features from the Gray-Level Co-occurrence Matrix (GLCM), thirteen features from the Gray-Level Run-Length Matrix (GLRLM), thirteen features from the Gray-Level Size Zone Matrix (GLSZM) and five features from the Neighborhood Gray-Tone Difference Matrix (NGTDM) using their open-source feature extraction codes (https://github.com/mvallieres/radiomics-develop). All the forty features were calculated under six different numbers of gray levels: fixed gray levels of 8, 16, 32, 64, 128, 512 sets of different parameters for each image. The isotropic voxel size for image resampling and feature extraction is 1mm. The image intensity quantization algorithm we used for feature extraction is uniform quantization algorithm which uniformly divides intensity range to fixed number of gray levels.

**Supplementary Table 2. Full list of texture radiomic features.**

| **Gray-Level Co-occurrence Matrix (GLCM)** | **Gray-Level Run-Length Matrix (GLRLM)** | **Gray-Level Size Zone Matrix (GLSZM)** | **Neighbourhood Gray-Tone Difference Matrix (NGTDM)** |
| --- | --- | --- | --- |
| Energy | Short Run Emphasis (SRE) | Small Zone Emphasis (SZE) | Coarseness |
| Contrast | Long Run Emphasis (LRE) | Large Zone Emphasis (LZE) | Contrast |
| Correlation | Gray-Level Nonuniformity (GLN) | Gray-Level Nonuniformity (GLN) | Busyness |
| Homogeneity | Run-Length Nonuniformity (RLN) | Zone-Size Nonuniformity (ZSN) | Complexity |
| Variance | Run Percentage (RP) | Zone Percentage (RP) | Strength |
| Sum Average | Run Percentage (RP) | Low Gray-Level Zone Emphasis (LGZE) |  |
| Entropy | High Gray-Level Run Emphasis (HGRE) | High Gray-Level Zone Emphasis (HGZE) |  |
| Dissimilarity | Short Run Low Gray-Level Emphasis (SRLGE) | Small Zone Low Gray-Level Emphasis (SZLGE) |  |
| Autocorrelation | Short Run High Gray-Level Emphasis (SRHGE) | Small Zone High Gray-Level Emphasis (SZHGE) |  |
|  | Long Run Low Gray-Level Emphasis (LRLGE) | Large Zone Low Gray-Level Emphasis (LZLGE) |  |
|  | Long Run High Gray-Level Emphasis (LRHGE) | Large Zone High Gray-Level Emphasis (LZHGE) |  |
|  | Gray-Level Variance (GLV) | Gray-Level Variance (GLV) |  |
|  | Run-Length Variance (RLV) | Zone-Size Variance (ZSV) |  |

For all the above mentioned 257 radiomics feature, we calculated the net-change of features before and after NAT as the treatment induced radiomic feature change, and we concatenated it with the pre-treatment radiomic features for constructing our delta-radiomics based treatment outcome prediction models. And we termed the concatenated baseline and delta-radiomics features as **BL-DRFs**.

1. **Workflow of feature selection and model construction**

The workflow of feature selection and model construction is shown in **Supplementary Figure S1**. We first performed 100 times 5-fold cross validation on the discovery cohort for pre-operative clinical feature, post-operative clinical features and BL-DRF pre-selection separately. The partition of training and validation is random in each time. Univariate cox regression models were built with each feature for overall survival (OS) and disease-free survival (DFS) prediction, univariate logistic regression models were built for tumor surgical margin (SM) binary prediction. The average C-index of univariate OS/DFS prediction models and AUC of univariate SM prediction on validation data were recorded, and the order of features was sorted based on the validation performance. To avoid the low-predictive-ability features, feature who has lower than 0.5 average AUC or C-index prediction performance was removed from the feature set corresponding to the prediction task. To reduce redundancy in the feature sets for different prediction targets, we performed Pearson correlation analysis for all the features. A feature that has an absolute correlation coefficient higher than 0.8 to any of its previous features was removed from the feature set for the corresponding prediction target.

Then, for prediction OS and DFS, we performed multivariate CPH regression with step forward feature selection strategy to further select predictive features and construct the survival models. The clinical models and BL-DRF models were built separately. C-index was the criteria for step forward feature selection in this step, 5 was set as the maximum number of selected clinical features, 10 was set as the maximum number of selected BL-DRFs. Another 100 times of 5-fold cross-validation were conducted to mitigate the impact of random patient partition, and the same partition were used in each time of 5-fold cross-validation for construct pre-clinical, post-clinical, and BL-DRF models. The risk scores of training samples and validation samples in each time of 5-fold cross-validation were recorded. The average validation C-indexes of pre- and post-operative models were used as the final survival prediction performance for pre- and post-operative OS/DFS models on internal cohort respectively. The risk scores of pre-operative clinical survival models and BL-DRF models on training data were then used again as two features for training the BL-DRF-PC models for OD/DFS prediction, and the average C-indexes on validation data were recorded as the BL-DRF-PC models’ performance on internal dataset. The average risk scores from the pre-operative clinical models, post-operative clinical models and BL-DRF-PC models were recorded and used to differentiate high- and low-risk patient group, the threshold was set as the median risk score.

As post-operative SM is not a time-related variable, we used a multi-classifier multi-objective binary outcome prediction model (mCOM) for the prediction. The model sets sensitivity and specificity as the objectives simultaneously, adopts support vector machine (SVM), logistic regression (LR) and discriminant analysis (DA) together to construct the model and fuses the output probabilities from single-modality models to give the final prediction. The proposed mCOM is optimized via an iterative multi-objective immune algorithm (IMIA). The optimization process including further feature selection, classifier parameter selection and weighting factor selection. For model details about mCOM model and IMIA, please refer to the **Supplementary Section D** and our previous work.^3,4^


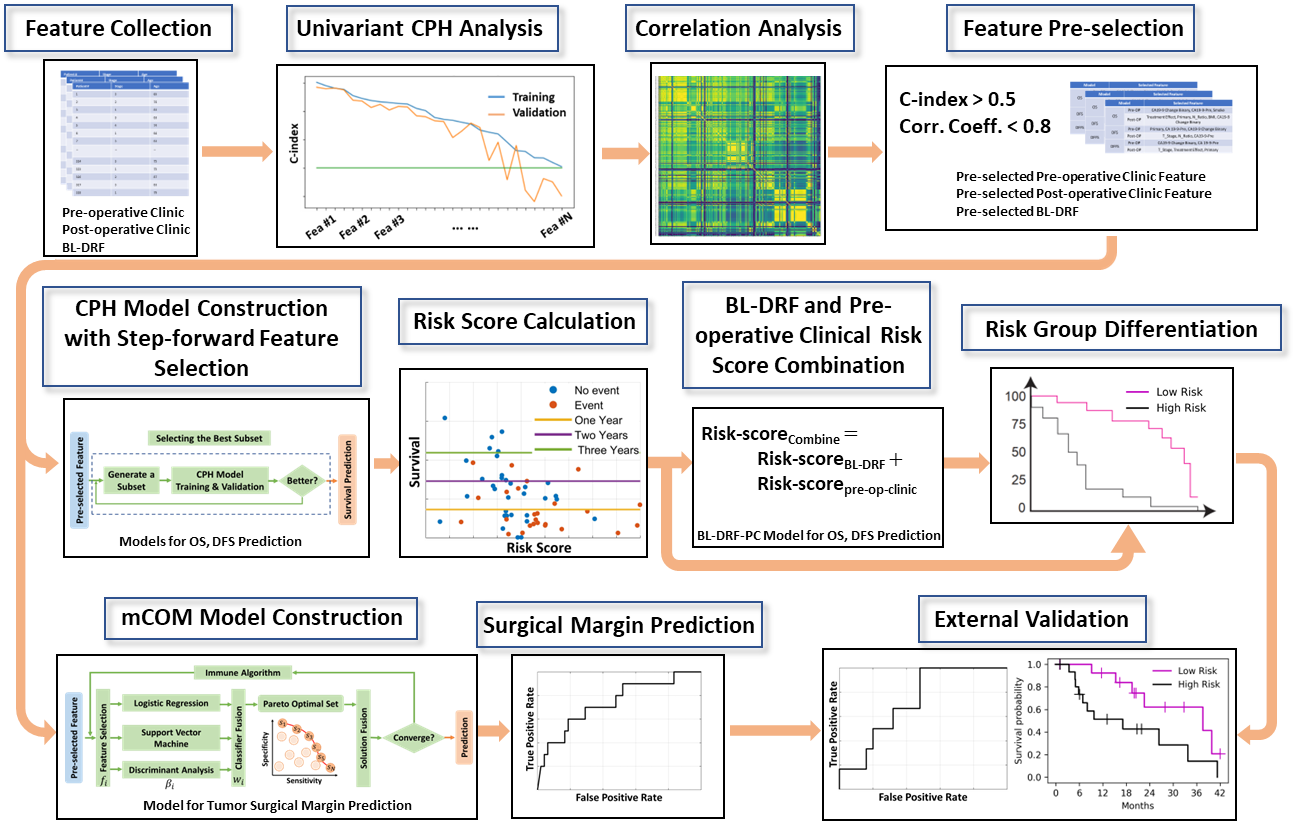


**Supplementary Figure 2.** Workflow of feature selection and model construction for survival prediction and surgery margin prediction.

1. **Details about mCOM model for surgical margin prediction**

We used multi-task multi-objective immune algorithm (mTO), to solve the objective function of mCOM model, which adopts both sensitivity and specificity as optimization targets. mTO consists of six steps: initialization, cloning, mutation, deletion, solution update and termination. In the initialization step, solution set $S$ is randomly initialized as $S\left( 0 \right)$, and $S\left( 0 \right)=\left\{ \theta_{1}, \ldots, \theta_{I_{0}} \right\}$, where $I_{0}$ is the number of solutions at the beginning of model training. Each individual solution $\theta_{i}, i= 1, 2, \ldots, I_{0},$ is defined as a group of parameters comprising feature selection vector $f_{i}$, classifier parameter vector $\beta_{i}$ and weighting factor vector $w_{i}.$ $f_{i}$ is a binary vector: a value of “1” indicates that the corresponding feature has been selected, while “0” indicates that it has not. $\beta_{i}$ is the vector containing all the parameters, including hyperparameters, of different classifiers, and $w_{i}$ is the weights used in classifier fusion to fuse the output probabilities of multiple classifiers into a single probability value.

After initialization, the first generation of solution sets for different modalities can be trained using features from training samples. A validation set is then used to evaluate all the solutions, and their performance—as measured by sensitivity, specificity, and AUC—is recorded and used as the basis for the solution cloning, mutation, and deletion operations in the next generation. In mTO, the cloning, mutation, deletion and solution updating operations are the same as the Iterative multi-objective immune algorithm (IMIA), the detailed implementation can be found in our previous work ^4,5^. When $t$ reaches the maximal number of generations $T$, the weights for solution fusion and the weights for modality fusion are calculated based on the recorded performance of solutions on the validation data, and then the algorithm terminates.

1. **Selected feature set for prognostic model construction**

We performed univariate feature analysis of the selected features in the final models. For survival models, the hazard ratios and univariate CPH model C-indexes calculated with the whole discovery cohort are listed. For SM prediction, the selected features and their corresponding univariate logistic regression model AUCs are recorded.

**Supplementary Table 3. Univariate analysis of the select features in the final models.**

| **Outcome** | **Feature** | **C-index (95% CI)** | **Hazard Ratio (95% CI)** | **p-value** |
| --- | --- | --- | --- | --- |
| OS | CA19-9 Pre | 0.49 | 1.22 (0.91, 1.64) | 0.17 |
|  | CA19-9 Change | 0.57 | 0.39 (0.18, 0.85) | 0.02 |
|  | GLCM Contrast 64 Delta | 0.64 | 0.68 (0.55, 0.85) | <0.01 |
|  | GLRLM GLN | 0.58 | 1.32 (1.01, 1.72) | 0.04 |
|  | GLCM Correlation 8 Delta | 0.61 | 1.23 (1.02, 1.48) | 0.03 |
|  | Minimum Intensity Delta | 0.57 | 0.89 (0.70, 1.14) | 0.36 |
| DFS | Resectability Status | 0.57 | 1.33 (0.86, 2.06) | 0.22 |
|  | CA19-9 Pre | 0.56 | 1.20 (0.92, 1.56) | 0.18 |
|  | CA19-9 Change | 0.57 | 0.41 (0.19, 0.90) | 0.03 |
|  | GLCM Correlation 8 | 0.65 | 1.57 (1.21, 2.03) | <0.01 |
|  | GLCM Homogeneity 64 Delta | 0.61 | 1.25 (1.00, 1.56) | 0.05 |
|  | NGTDM Contrast 16 | 0.60 | 1.34 (0.90, 1.98) | 0.15 |

1. **Performance comparison of different models for survival prediction and surgical margin status prediction**

In the proposed models, we used the conjunction of baseline radiomics features (pre-NAT features) and net change of radiomics features between features extracted from images acquired before and after NAT (delta-radiomics features) as radiomics feature input. Not only in this work, but in our previous study, we found that constructing a model using delta-radiomics features alone does not provide superior predictive capabilities when used independently from the baseline radiomics feature, and this agrees with other published work who also termed their final model ‘delta-radiomics-based’.^6,7^ Based on this, we concatenated pre-treatment radiomics features and delta-radiomics features as the radiomics feature input (BL-DRF) for training the radiomics model of each imaging modality. For comparison, we also trained radiomics models based on relative delta-radiomics features, where the relative delta-radiomics features are the ratios between feature values change and pre-treatment feature values ($DRF_{relative}=\frac{Feature_{post}-Feature_{pre}}{Feature_{pre}}$).

To show the difference in predictive abilities of models built with delta-radiomics only (net change radiomics features) and BL-DRFs, we summarized the prediction performance of models built with delta-radiomics only and compared with that of the BL-DRF models for OS prediction and DFS prediction using UTSW dataset (5-fold CV) in Supplementary Table 4.

**Supplementary Table 4.** Comparison of prediction performance of models built with different feature sets. Overall survival (OS) and disease-free survival (DFS) prediction are evaluated with C-index (95% confidence interval), resection margin status (RMS) prediction is evaluated with AUC. Feature sets used here comprise different modalities: pre-operative clinical feature, post-operative clinical feature, pre-neoadjuvant treatment (NAT) radiomics feature (Pre-NAT), post-NAT radiomics feature (Post-NAT), delta-radiomic feature (DRF), combination of baseline feature and DRF (BL-DRF), combination of baseline features and relative-DRF (BL-Relative-DRF), combined BL-Relative-DRF and pre-operative clinical feature (BL-Relative-DRF-PC), and combined BL-DRF and pre-operative clinical feature (BL-DRF-PC).

| **Supplementary Table 4**  **Discovery Cohort** | | | | | | | | | | | |
| --- | --- | --- | --- | --- | --- | --- | --- | --- | --- | --- | --- |
| Modality | Feature | OS (95% CI) | *P*-value | DFS (95% CI) | *P*-value | RMS (mCOM) | | *P*-value | |  |  |
| Clinical | Pre-operative Clinical | 0.625 (0.617, 0.633) | <0.01^§^ | 0.589 (0.581, 0.598) | <0.01^§^ | 0.59 | | <0.01^§^ | |  |  |
|  | Post-operative Clinical | 0.719 (0.713, 0.726) | 0.05^§^ | 0.662 (0.656, 0.668) | <0.01^§^ | 0.68 | | 0.03^§^ | |  |  |
| Radiomics | Pre-NAT | 0.615 (0.610, 0.621) | <0.01^*^ | 0.610 (0.605, 0.614) | <0.01^*^ | 0.65 | | <0.01^*^ | |  |  |
|  | Post-NAT | 0.638 (0.633, 0.643) | <0.01^*^ | 0.706 (0.700, 0.710) | <0.01^*^ | 0.69 | | 0.04 | |  |  |
|  | DRF | 0.612 (0.605, 0.619) | <0.01^*^ | 0.642 (0.636, 0.648) | <0.01^*^ | 0.60 | | <0.01^*^ | |  |  |
|  | BL-DRF | 0.722 (0.716, 0.729) | 0.21^§^ | 0.739 (0.731, 0.741) | 0.13^§^ | 0.73 | | 0.85^§^ | |  |  |
|  | BL-Relative-DRF | 0.665 (0.623, 0.679) | <0.01^*^ | 0.689 (0.667, 0.712) | <0.01^*^ | 0.60 | | <0.01^*^ | |  |  |
| Combined | BL-Relative-DRF-PC | 0.711 (0.703, 0.720) | 0.03^§^ | 0.703 (0.695, 0.726) | <0.01^§^ | 0.69 | | 0.05^§^ | |  |  |
|  | BL-DRF-PC | **0.732** (0.721, 0.736) | ---- | 0.745 (0.736, 0.752) | ---- | **0.73** | | ---- | |  |  |
| **Validation Cohort** | | | | | | | | | | | |
| Modality | Feature | OS (95% CI) | *P*-value | DFS (95% CI) | *P*-value | | RMS (mCOM) | | *P*-value | |  |
| Clinical | Pre-operative Clinical | 0.631 (0.623, 0.638) | <0.01^$^ | 0.590 (0.585, 0.595) | <0.01^$^ | | 0.62 | | 0.05^$^ | |  |
|  | Post-operative Clinical | 0.650 (0.647, 0.653) | <0.01^$^ | 0.707 (0.707, 0.708) | <0.01^$^ | | 0.65 | | 0.62^$^ | |  |
| Combined | BL-Relative-DRF | 0.652 (0.641, 0.656) | <0.05^$^ | 0.610 (0.597, 0.623) | <0.01^$^ | | 0.64 | | 0.23^$^ | |  |
|  | BL-DRF-PC | **0.663** (0.656, 0.668) | ---- | 0.644 (0.640, 0.653) | ---- | | **0.69** | | ---- | |  |

^*^ *P*-values were compared with concatenated baseline- and delta-radiomics feature (BL-DRF) model on discovery cohort.

^§^ P-values were compared with combined BL-DRF and pre-operative clinical (BL-DRF-PC) model on validation cohort.

^$^ P-values were compared with BL-DRF-PC model on validation cohort.

Paired-t-test was used for comparing OS/DF C-index, Delong’s test was used for comparing TMS prediction ROCs.

For resection margin status (RMS) prediction, in addition to different feature sets presented in the above table, we also compared our multi-classifier multi-objective binary outcome prediction method (mCOM) to two baseline models widely used in radiomics studies: 1) logistic regression model, 2) support vector machine model ^8-11^. Least absolute shrinkage and selection operator (LASSO) method was used for feature selection for these two models, models constructed with pre-operative clinical feature only, delta-radiomics feature only, and combined clinical and delta-radiomics were evaluated for comparison.

**Supplementary Table 5.** Comparison of logistic regression, support vector machine, and proposed multi-classifier multi-objective binary outcome prediction model (mCOM) for resection margin status (RMS) prediction. The performance is evaluated with AUC. Feature sets used here comprise pre-neoadjuvant treatment (NAT) radiomics features (Pre-NAT), delta-radiomics features (DRF), combination of baseline feature and DRF (BL-DRF), pre-operative clinical features, and combination of BL-DRF and pre-operative clinical features (BL-DRF-PC).

| **Supplementary Table 5**  **Discovery Cohort** | | | | |
| --- | --- | --- | --- | --- |
| Modality | Feature | mCOM | Logistic Regression | Support Vector Machine |
| Radiomics | Pre-NAT | 0.65 | 0.62 | 0.64 |
|  | DRF | 0.60 | 0.58 | 0.59 |
|  | BL-DRF | 0.73 | 0.65 | 0.65 |
| Clinical | Pre-operative Clinical | 0.59 | 0.60 | 0.59 |
| Combined | BL-DRF-PC | **0.73** | 0.66 | 0.68 |
| **Validation Cohort** | | | | |
| Modality | Feature | mCOM | Logistic Regression | Support Vector Machine |
| Clinical | Pre-operative Clinical | 0.62 | 0.58 | 0.63 |
| Combined | BL-DRF-PC | **0.69** | 0.61 | 0.65 |

1. **Binary prediction results using risk score as the only criteria**

To evaluate the ability of predict binary outcomes at different follow-ups using the risk scores generated from our survival prediction models, we used the combined risk score as the only criteria to identify whether a patient is of high risk of death or cancer recurrence at the time points of 1, 2 and 3 years after surgery. Patients who didn’t have enough follow-up were excluded for evaluating the prediction performance of the binary prediction models of the corresponding time points. The prediction performance is listed in Table S6.

**Supplementary Table 6.** Prediction performances when risk scores from survival prediction models were used to differentiate different outcomes at the time points of 1, 2 and 3 years on discovery cohort. #Positive means number of positive samples, so as #Negative.

| Outcome | Metrics | One-year | Two-year | Three-year |
| --- | --- | --- | --- | --- |
| Patient Death | #Positive | 3 | 22 | 34 |
|  | #Negative | 55 | 35 | 12 |
|  | Sensitivity | 0.67 | 0.82 | 0.74 |
|  | Specificity | 0.44 | 0.60 | 0.75 |
|  | Accuracy | 0.45 | 0.68 | 0.74 |
|  | AUC | 0.74 | 0.79 | 0.84 |
| Cancer Recurrence | #Positive | 25 | 30 | 33 |
|  | #Negative | 27 | 13 | 6 |
|  | Sensitivity | 0.80 | 0.73 | 0.70 |
|  | Specificity | 0.67 | 0.69 | 0.69 |
|  | Accuracy | 0.73 | 0.72 | 0.69 |
|  | AUC | 0.86 | 0.81 | 0.84 |

1. **Evaluation of radiomics score of this study**

We evaluated the reliability and reproducibility of our methodology with a modified radiomics study quality score.^12,13^ Based on the criteria suggested, we got 16/36 now (median score for the reviewed works is 7/36).^12,13^

**Supplementary Table 7.** Checklist of radiomics study quality score (mRQS) for evaluating the methodology of the presenting work.

| Criteria | | Points | Our Points & Reason |
| --- | --- | --- | --- |
| 1 | Image protocol quality –well- documented image protocols and/or usage of public image protocols allow reproducibility/replicability | +1 if protocols are well documented  +1 if public protocol is used | +1 |
| 2 | Multiple segmentation – possible actions are: segmentation by different physicians/algorithms/software, perturbing segmentations by (random) noise, segmentation at different breathing cycles. Analyse feature robustness to segmentation variabilities. | +1 | 0 |
| 3 | Phantom study on all scanners – detect inter-scanner differences and vendor dependent features. Analyse feature robustness to these sources of variability | + 1 *This criteria is only  applicable to radiology based  approaches (both radiomics  based and non-radiomics  based) | 0 |
| 4 | Imaging at multiple time points - collect images of individuals at additional time points. Analyse feature robustness to temporal variability (for example, organ movement, organ expansion/ shrinkage) | + 1 *This criteria is only  applicable to radiology  approaches (both radiomics  based and non-radiomics  based) | 0 |
| 5 | Feature reduction or adjustment for multiple testing - decreases the risk of overfitting. Overfitting is inevitable if the number of features exceeds the number of samples. Consider feature robustness when selecting features | - 3 (if neither measure is  implemented) +3 (if either measure is implemented) *This criteria is only applicable to radiomics based  radiology approaches | +3 |
| 6 | Multivariable analysis with non AI based pathology/radiology imaging features (for example, EGFR mutation) - is expected to provide a more holistic model. Permits correlating/inferencing between AI-based pathology/radiology imaging and non AI-based pathology/radiology imaging features | +1 | 0 |
| 7 | Detect and discuss biological correlates - demonstration of phenotypic differences (possibly associated with underlying gene–protein expression patterns) deepens understanding of pathology/radiology based imaging and biology | +1 | 0 |
| 8 | Cut-off analyses - determine risk groups by either the median, a previously published cut-off or report a continuous risk variable. Reduces the risk of reporting overly optimistic results | + 1 *This criteria is only  applicable to radiomics based radiology approaches | +1 |
| 9 | Discrimination statistics - report  discrimination statistics (for example,  C-statistic, ROC curve, AUC) and their  statistical significance (for example, p  values, confidence intervals). One can  also apply resampling method (for  example, bootstrapping, cross  validation) | + 1 (if a discrimination  statistic and its statistical  significance are reported) +1  (if a resampling method  technique is also applied) | +2 |
| 10 | Calibration statistics - report calibration  statistics (for example, Calibration-in  the-large/slope, calibration plots) and  their statistical significance (for  example, P-values, confidence  intervals). One can also apply  resampling method (for example,  bootstrapping, cross-validation) | + 1 (if a calibration statistic  and its statistical significance  are reported) +1 (if a  resampling method technique  is also applied) | +1 |
| 11 | Prospective study registered in a trial  database - provides the highest level of  evidence supporting the clinical  validity and usefulness of the  pathology/radiology biomarker | + 7 (for prospective validation  of a pathology/radiology based  imaging approach in an  appropriate trial) | 0 |
| 12 | Validation - the validation is performed  without retraining and without  adaptation of the cut-off value,  provides crucial information with  regard to credible clinical performance | - 5 (if validation is missing) +2  (if validation is based on a  dataset from the same  institute) +3 (if validation is  based on a dataset from  another institute) +4 (if  validation is based on two  datasets from two distinct  institutes) +4 (if the study  validates a previously  published AI based  pathology/radiology | +3 |
| 13 | Comparison to ‘gold standard’ - assess  the extent to which the model agrees  with/is superior to the current ‘gold  standard’ method (for example, TNM  staging for survival prediction). This  comparison shows the added value of  AI based pathology/radiology imaging  approaches | +2 | +2 |
| 14 | Potential clinical utility - report on the  current and potential application of the  model in a clinical setting (for  example, decision curve analysis) | +2 | +2 |
| 15 | Cost-effectiveness analysis - report on  the cost-effectiveness of the clinical  application (for example, QALYs  generated) | +1 | +1 |
| 16 | Cost-effectiveness analysis - report on  the cost-effectiveness of the clinical  application (for example, QALYs  generated) | + 1 (if scans are open source)  + 1 (if region of interest  segmentations are open  source) + 1 (if code is open  source) + 1 (if AI-based  pathology/radiology features  are calculated on a set of  representative ROIs and the  calculated features and  representative ROIs are open  source) | 0 |

**Supplementary Table 8.** Checklist for evaluation of radiomics research (CLEAR) for our study.

| Section | No. | Item | Yes | No | n/a | Page |
| --- | --- | --- | --- | --- | --- | --- |
| Title |  |  |  |  |  |  |
|  | 1 | Relevant title, specifying the radiomic methodology  Indicate the use of radiomics in the title. The following details can also be considered to be specified in the title: radiomic technique (e.g., hand-crafted, engineered, deep, delta, etc.), modality (e.g., computed tomography [CT], magnetic resonance imaging [MRI], ultrasound), important aspects of the scans (e.g., unenhanced, dynamic), use of machine learning (e.g., machine learning-based), external validation, and multi-center design. | ☑ | ☐ | ☐ | Title |
| Abstract |  |  |  |  |  |  |
|  | 2 | Structured summary with relevant information  Provide a structured summary of the purpose, methods, results, and conclusions, presenting only the most important aspects directly related to the purpose of the study. The abstract should be understandable on its own, without reading the main text. Considering the submission guidelines of the journals, it is recommended to specify the following items: the baseline characteristics (e.g., number of patients, scans, images, classes), data source (e.g., public, institutional), study nature (e.g., prospective, retrospective), segmentation technique (e.g., automated, semi-automated, or manual), feature extraction technique (e.g., hand-crafted, engineered, deep), dimensionality reduction techniques (e.g., feature selection, reproducibility analysis, multi-collinearity), modeling details (e.g., algorithms/models), validation technique (e.g., cross-validation), unseen testing (internal hold-out, external testing), model performance metrics (e.g., the area under the curve) with uncertainty measures (e.g., confidence intervals), number of the final set of features, traditional statistical methods with p-values, and open science status (e.g., public availability of data, code, and/or model). | ☑ | ☐ | ☐ | Abstract |
| Keywords |  |  |  |  |  |  |
|  | 3 | Relevant keywords for radiomics  List the primary keywords that indicate (e.g., radiomics, texture analysis) and characterize a radiomic study (e.g., machine learning, deep learning, computed tomography, magnetic resonance imaging, reproducibility), unless the journal requires exclusive use of certain terms (e.g., MeSH terms, which do not yet include radiomics-specific terms). | ☑ | ☐ | ☐ | Keywords |
| Introduction |  |  |  |  |  |  |
|  | 4 | Scientific or clinical background  Define the scientific or clinical problem with a summary of the related literature and knowledge gaps, including a short review of the current state of knowledge. Describe why the scientific question is technically or clinically important. | ☑ | ☐ | ☐ | 1-2 |
|  | 5 | Rationale for using a radiomic approach  Describe why a radiomic approach is considered. Performance and problematic aspects of currently used methods need to be described. Mention what the radiomics approach would offer to solve these problems. Clearly state how radiomics could affect clinical practice considering the study question. | ☑ | ☐ | ☐ | 1-2 |
|  | 6 | Study objective(s)  Describe the purpose of the study while focusing on the scientific problem. Mention the expected contributions to the current literature. | ☑ | ☐ | ☐ | 2 |
| Method |  |  |  |  |  |  |
| *Study Design* | 7 | Adherence to guidelines or checklists (e.g., CLEAR checklist)  Indicate that the CLEAR checklist was used for reporting and submit the checklist as supplemental data. Do the same with other checklists or guidelines if used in addition to the CLEAR checklist. | ☑ | ☐ | ☐ | 3, Supplementary |
|  | 8 | Ethical details (e.g., approval, consent, data protection)  Describe the ethical questions to ensure that the study was conducted appropriately. Give information about ethical approval, informed consent, and data protection (e.g., de-identification) if the data is from private sources. | ☑ | ☐ | ☐ | 3 |
|  | 9 | Sample size calculation  Describe how the sample size or power was determined before or after the study (e.g., sample size/power calculation, based on availability). | ☐ | ☑ | ☐ |  |
|  | 10 | Study nature (e.g., retrospective, prospective)  Indicate whether the study is prospective or retrospective and case/control or cohort, etc. In the case of prospective studies, provide registration details if available. | ☑ | ☐ | ☐ | 3 |
|  | 11 | Eligibility criteria  Define the inclusion criteria first. Then, specify the exclusion criteria. Avoid redundancies by using the opposite of the inclusion criteria as exclusion criteria. Specify the selection process (e.g., random, consecutive). Keep the numeric details of eligibility for the results. | ☑ | ☐ | ☐ | 3, Figure 2 |
|  | 12 | Flowchart for technical pipeline  Provide a flowchart for summarizing the key methodological steps in the study. Due to the complex nature of the radiomic approaches, such flowcharts help readers better understand the methodology. | ☑ | ☐ | ☐ | Figure 1 |
| *Data* | 13 | Data source (e.g., private, public)  State the data source (e.g., private, public, mixed [both private and public]). State clearly which data source is used in different data partitions. Provide web links and references if the source is public. Give the image or patient identifiers as a supplement if public data is used. | ☑ | ☐ | ☐ | 3 |
|  | 14 | Data overlap  State if any part of the dataset was used in a previous publication. Describe the differences between the current study and previous studies in terms of study purpose and methodology. | ☐ | ☐ | ☑ |  |
|  | 15 | Data split methodology  Describe the data split into training, validation, and test sets. Mention that multiple splits are created (e.g., k-fold cross-validation or bootstrapping). Specify how the assignment was done (e.g., random, semi-random, manual, center-wise, chronological order). Indicate the ratio of each partition, with class proportions. Describe at which level the data is split (e.g., patient-wise, image-wise, study-wise, scanner-wise, institution-wise). Clearly state the measures undertaken to avoid information leakage across datasets (e.g., creating the hold-out test set before feature normalization, feature selection, hyperparameter optimization, and model training) [23]. Note that any test data should only be used once for evaluation of the final model to prevent optimistic biases. Declare the systematic differences among the data partitions. | ☑ | ☐ | ☐ | 3, 5 |
|  | 16 | Imaging protocol (i.e., image acquisition and processing)  Provide the imaging protocol and acquisition parameters with post-processing details. Define physical pixel and voxel dimensions. Clearly state whether single or multiple or various scanners are used, with the number of instances for each protocol. Define the timing of the phase if a contrast medium was used. State the patient preparation (drug administration, blood sugar control before the scans, etc.) if performed. | ☑ | ☐ | ☐ | 3, Supplementary |
|  | 17 | Definition of non-radiomic predictor variables  Describe the data elements appearing as non-radiomic predictors. Non-radiomic variables might be demographic characteristics (e.g., age, gender, ethnicity), widely used traditional laboratory biomarkers (e.g., carcinoembryonic antigen), or traditional approaches used in daily clinical practice (e.g., radiologist’s qualitative reading, Hounsfield Unit evaluation, Response Evaluation Criteria in Solid Tumors [RECIST], Response Assessment in Neuro-Oncology [RANO] criteria). It would be helpful to know how these predictors were identified (e.g., based on a literature review). If applicable, describe any transformation of predictors (e.g., binarization of continuous predictors, the grouping of levels of categorical variables). | ☑ | ☐ | ☐ | 5 |
|  | 18 | Definition of the reference standard (i.e., outcome variable)  Describe the reference standard or outcome measure that the radiomic approach will predict (e.g., pathological grade, histopathological subtypes, genomic markers, local-regional control, survival, etc.). Provide the rationale for the choice of the reference standard (e.g., higher reproducibility rates). Clearly state the reproducibility concerns, potential biases, and limitations of the reference standard. | ☑ | ☐ | ☐ | 2 |
| *Segmentation* | 19 | Segmentation strategy  Indicate which software programs or tools are used for segmentation or annotation. Specify the version of the software and the exact configuration parameters. Provide reference and web link to the software. Describe the segmentation method (e.g., automatic, semi-automatic, manual). Provide the rules of the segmentation (e.g., margin shrinkage or expansion from the visible contour, included/excluded regions). Provide figures to show the segmentation style. Provide image registration details (e.g., software, version, link, parameters) if segmentation is propagated for multi-modal (e.g., CT and MR), multi-phase (e.g., unenhanced, arterial, venous phase CT), or multi-sequence (e.g., T2-weighted, post-contrast T1-weighted, diffusion-weighted imaging) analyses. If radiomic features are extracted from 2D images on a single slice, please explain with which criteria the slice is chosen. In the case of several lesions, explain if all the lesions are segmented and describe how the feature values are aggregated. If only one lesion is chosen, describe the criteria (e.g., the primitive or the most voluminous). | ☑ | ☐ | ☐ | 3 |
|  | 20 | Details of operators performing segmentation  State how many readers performed the segmentation, as well as their experience. In the case of multiple readers, describe how the final form of segmentation is achieved (e.g., the consensus of readers, intersection of segmentations, independent segmentation for further reproducibility analysis, sequential refinements from numerous expert raters until convergence), which is particularly important for the training data because the segmentation process on the test data should be as close to the clinical practice as possible, that is, the segmentation of a single reader. | ☑ | ☐ | ☐ | 3 |
| *Pre-processing* | 21 | Image pre-processing details  Indicate which software programs or tools are used for pre-processing. Specify the version of the software and the exact configuration parameters. Provide reference and web link to the software, if available. Describe all pre-processing techniques and associated parameters applied to the image including the normalization (e.g., minimum-maximum normalization, standardization, logarithmic transformation, bias field correction), de-noising, skull stripping (also known as brain extraction), interpolation to create uniform images (e.g., in terms of slice thickness), standardized uptake value conversion, and registration. Also, state if an image or feature-based harmonization technique was used. | ☑ | ☐ | ☐ | 4 |
|  | 22 | Resampling method and its parameters  Specify the resampling technique (e.g., linear, cubic b-spline) applied to the pixels or voxels. Provide the physical pixel and voxel dimensions after resampling. | ☑ | ☐ | ☐ | 4, Supplementary |
|  | 23 | Discretization method and its parameters  Specify the discretization method (e.g., fixed bin width, fixed bin count method, or histogram equalization) used for hand-crafted radiomic feature extraction. Report the rationale for using a particular discretization technique. Indicate the number of grey levels for the fixed bin count method or the bin width as well as the value of the first level (or minimum and maximum bounds) for the fixed bin width method. Any experimental detail with different discretization methods and values is important to declare. | ☑ | ☐ | ☐ | Supplementary |
|  | 24 | Image types (e.g., original, filtered, transformed)  Provide the image types from which the radiomic features are extracted, e.g., original or images with convolutional filters (e.g., Laplacian of Gaussian edge enhancement, wavelet decomposition) [24]. Also, give nuances about the parameters of transformed image types (e.g., sigma values of Laplacian of Gaussian filtering). | ☑ | ☐ | ☐ | 3 |
| *Feature extraction* | 25 | Feature extraction method  Indicate which software programs or tools are used for radiomic feature extraction. Specify the version of the software and the exact configuration parameters (also see Item#55). Provide reference and web link to the software. Indicate if the software adheres to the benchmarks/certification of IBSI [25]. Specify the general feature types, such as deep features, hand-crafted features, engineered features, or a combination. Refer to the mathematical formulas of the hand-crafted and engineered features. Provide formulas and code if new hand-crafted features are introduced. Present the architectural details for deep feature extraction. Provide details of any feature engineering performed. Specify whether radiomic features are extracted in a two-dimensional (2D) plane, 2D tri-planar, or three-dimensional (3D) space. If 2D features are extracted from 3D segmentation, provide reasons (e.g., large slice thickness) as to why such an approach is followed. | ☑ | ☐ | ☐ | 4, Supplementary |
|  | 26 | Feature classes  Provide the radiomic feature classes (e.g., shape, first-order, grey-level co-occurrence matrix). Use IBSI terminology for feature classes [25]. Specify the number of features per feature class. Mention if any feature class is excluded with reason. | ☑ | ☐ | ☐ | 4 |
|  | 27 | Number of features  Indicate the total number of features per instance. If applicable, provide the number of features per imaging modality and its components (e.g., phase for CT, sequence for MRI, etc.). | ☑ | ☐ | ☐ | 4, Supplementary |
|  | 28 | Default configuration statement for remaining parameters  After providing all modified parameters of pre-processing and radiomic feature extraction, state clearly that all other parameters remained as a default configuration. | ☑ | ☐ | ☐ | Supplementary |
| *Data preparation* | 29 | Handling of missing data  State if, and how much, missing data are present in the study. If so, provide details as to how it was addressed (e.g., deletion, substitution, or imputation). | ☐ | ☐ | ☑ |  |
|  | 30 | Details of class imbalance  Indicate the balance status of the classes according to the reference standard. Provide details about how class imbalance is handled. Specify the techniques (e.g., synthetic minority over-sampling, simple over-sampling through replication, under-sampling) used to achieve the class balance. Clearly state these data augmentation and under-sampling strategies are applied only in the training set. | ☑ | ☐ | ☐ | 9, Table 1 |
|  | 31 | Details of segmentation reliability analysis  Describe the reliability analysis done to assess the influence of segmentation differences. An intra- and inter-rater reproducibility analysis must be considered in manual and semi-automatic methods. Provide details about the statistical tests used for the reliability analysis (e.g., intraclass correlation coefficient along with types) [26]. Mention the independence of assessment. Clearly state the reliability analysis is performed using the training set only. | ☑ | ☐ | ☐ | 3-4 |
|  | 32 | Feature scaling details (e.g., normalization, standardization)  If applicable, describe the normalization technique applied to the radiomic feature data (e.g., minimum-maximum normalization, standardization, logarithmic transformation, ComBat normalization [choice of the batch, parametric or not, with or without empirical Bayes]). Specify the normalization scale. It is important to emphasize that this procedure is applied to the numeric radiomic feature data, not the images, in the training set and independently applied to the validation and test sets. | ☑ | ☐ | ☐ | Supplementary |
|  | 33 | Dimension reduction details  Specify the dimension reduction methods used, if applicable (e.g., collinearity analysis, reproducibility analysis, algorithm-based feature selection). Provide details about the statistical methods used. For example, provide the relevant statistical cut-off values for each step (e.g., features with intraclass correlation coefficient ≤0.9 are excluded). Clearly state the dimension reduction that is performed using the training set. Specify how the final number of features is achieved, for instance, the “rule of thumb” of ten features maximum for each instance. | ☑ | ☐ | ☐ | Supplementary |
| *Modeling* | 34 | Algorithm details  Provide the name and version of software programs or packages used for modeling. Refer to the related publication of the software if available. Specify the algorithms used to create models with architectural details including inputs, outputs, and all intermediate components. The description of the architecture should be complete to allow for exact replication by other investigators (also see Item#55 and Item#56). When a previously described architecture is used, refer to the previous work and specify any modification. If the final model involved an ensemble of algorithms, specify the type of ensemble (e.g., stacking, majority voting, averaging, etc.). | ☑ | ☐ | ☐ | 5-7, Supplementary |
|  | 35 | Training and tuning details  Describe the training process with adequate detail. Specify the augmentation technique, stopping criteria for training, hyperparameter tuning strategy (e.g., random, grid-search, Bayesian), range of hyperparameter values used in tuning, optimization techniques, regularization parameters, and initialization of model parameters (e.g., random, transfer learning). If transfer learning is applied, clearly state which layers or parameters are frozen or affected. | ☑ | ☐ | ☐ | 6-7 |
|  | 36 | Handling of confounders  Describe the method (e.g., directed acyclic graphs) for the detection of potential confounders (e.g., differences in tumor size between cohorts, different image acquisition parameters such as slice thickness, and differences in patient populations between primary and secondary hospitals) [27, 28]. Please describe how confounding was addressed (e.g., covariate adjustment). | ☐ | ☑ | ☐ |  |
|  | 37 | Model selection strategy  Describe how the final model was selected. Two broad categories for these are probabilistic (e.g., Akaike information criterion, Bayesian information criterion) and resampling methods (e.g., random train-test split, cross-validation, bootstrap validation) [12, 29]. Clearly state that only the training and validation sets are used for model selection. State if the model complexity is considered in selection, for instance, the “one standard error rule” [30]. Specify which performance metrics were used to select the final model. | ☑ | ☐ | ☐ | 5-7, Supplementary |
| *Evaluation* | 38 | Testing technique (e.g., internal, external)  Clearly state whether the model was internally or externally tested. The term “external testing” should only be used for the process that involves data usage from different institutions. In the case of external testing, specify the number of sites providing data and further details about whether they are used for multiple testing or in a single test. Describe the data characteristics and state if there are any differences among training, validation, internal testing, and external testing datasets (e.g., different scanners, different readers for segmentation, different ethnicity). Again, note that any test data should only be used once for evaluation to prevent biased performance metric estimates. | ☑ | ☐ | ☐ | 7 |
|  | 39 | Performance metrics and rationale for choosing  Specify the performance metrics to evaluate the predictive ability of the models. Justify the selected metrics according to the characteristics of the data (e.g., class imbalance). Beware of the potential pitfalls and follow recommendations when selecting the appropriate performance metrics [7, 31]. | ☑ | ☐ | ☐ | 7 |
|  | 40 | Uncertainty evaluation and measures (e.g., confidence intervals)  Describe the uncertainty evaluation (e.g., robustness, sensitivity analysis, calibration analysis if applicable) and measures of uncertainty quantification (e.g., confidence intervals, standard deviation). | ☑ | ☐ | ☐ | 6-7 |
|  | 41 | Statistical performance comparison (e.g., DeLong’s test)  Specify the statistical software and version used. Indicate which method was used for the comparison of the model performance such as the DeLong’s test [32, 33], McNemar’s test [34], or Bayesian approaches [35]. Provide a statistical threshold for the comparison (e.g., p<0.05) along with confidence intervals if applicable to the method or metric. Also, state if multiplicity is considered and corrected when comparing multiple models (e.g., p-value adjustment, Bonferroni correction, false-discovery rate). Report threshold values to stratify data into groups for statistical testing (e.g., the operating point on the receiver operating characteristic [ROC] curve to define the confusion matrix, and cut-off values for defining strata in survival analysis). | ☑ | ☐ | ☐ | 7 |
|  | 42 | Comparison with non-radiomic and combined methods  Indicate whether comparisons with non-radiomic approaches (e.g., clinical parameters, laboratory parameters, traditional radiological evaluations) are performed. Non-radiomic approaches can be combined with radiomic data as well (e.g., clinical-radiomic evaluation). Explain how the clinical utility is assessed, such as with decision curve analysis [36]. | ☑ | ☐ | ☐ | 5, 9, Supplementary |
|  | 43 | Interpretability and explainability methods  Describe the techniques used to increase the interpretability and explainability of the models created, if applicable [37]. Figures (e.g., class activation maps, feature maps, SHapley Additive exPlanations, accumulated local effects, partial dependence plots, etc.) related to the interpretability and explainability of the proposed radiomic model can be provided. | ☑ | ☐ | ☐ | 10, 12-13 |
| Results |  |  |  |  |  |  |
|  | 44 | Baseline demographic and clinical characteristics  Provide the baseline demographic, clinical, and imaging characteristics in text and/or tables. Report the information separately for training, validation (i.e., cross-validation), and test datasets, along with grouping based on the reference standard or non-radiomic variables. Associated statistical tests should also be provided to identify if the sets are identical or not. Provide whether any confounder is detected and handled appropriately. | ☑ | ☐ | ☐ | Table 1 |
|  | 45 | Flowchart for eligibility criteria  Provide a flowchart for summarizing eligibility criteria with the number of included and excluded instances. If more than one data source is involved, please give details for each source separately. | ☑ | ☐ | ☐ | Figure 2 |
|  | 46 | Feature statistics (e.g., reproducibility, feature selection)  Give statistical information (e.g., distribution of features based on outcome variables) of the selected features for inclusion into the model. Provide the name and number of reproducible features (e.g., for segmentation reproducibility, for reproducibility against image perturbations). Create a table for the selected features with details of feature name, class, and image type. Also, provide results of reproducibility statistics. Reproducibility metrics of selected features can be presented in tables or supplementary files. Figures (e.g., boxplots, correlation matrix, feature importance plots) and tables of descriptive summaries of features can be provided. | ☐ | ☑ | ☐ |  |
|  | 47 | Model performance evaluation  Provide the performance metrics for training, validation (e.g., multiple splits like cross-validation, bootstrapping, etc.), and unseen test data, separately. A summary of the most important findings should be given in the text. Provide the ‘no information rate’ as well. Details can be provided in figures (e.g., ROC curves, precision-recall curves) and tables. It is a good practice to provide figures for calibration statistics to show the robustness of model performance. Present additional figures to showcase examples of true and false predictions to help readers better understand the strengths and limitations of the proposed strategy. | ☑ | ☐ | ☐ | 10-11, Supplementary |
|  | 48 | Comparison with non-radiomic and combined approaches  Give the results about the comparison of radiomic approaches with non-radiomic (e.g., visual analysis, clinical only parameters) or combined approaches in the text and preferably on a table. Present the results for training, validation, and test data, separately. Provide uncertainty measures (e.g., confidence intervals, standard deviation, etc.) and statistical comparison results with p-values for each. Confusion matrices must also be provided. Aside from the predictive performance, specify which model is superior to others in terms of clinical utility. The clinical utility can be presented with a decision curve analysis. For the decision curve analysis, quantify the net benefit according to optimal probability thresholds, with multiple cut-points associated with different clinical views. Also, provide the rationale for why a specific threshold could be appropriate and clearly state what is meant by all and none strategies. | ☑ | ☐ | ☐ | 10-11, Supplementary |
| Discussion |  |  |  |  |  |  |
|  | 49 | Overview of important findings  Provide a summary of the work and an overview of the most important findings. No statistical information is needed. Try to position the study into one of the following categories: proof of concept evaluation, technical task-specific evaluation, clinical evaluation, and post-deployment evaluation [38]. Summarize the contribution to the literature. | ☑ | ☐ | ☐ | 12-14 |
|  | 50 | Previous works with differences from the current study  Provide the most important and relevant previous works. Mention the most prominent differences between the current study and the previous works. | ☑ | ☐ | ☐ | 12-14 |
|  | 51 | Practical implications  Summarize the practical implications of the results. Describe the key impact of the work on the field. Highlight the potential clinical value and role of the study. Discuss any issues that may hamper the successful translation of the study into real-world clinical practice. Also, provide future expectations and possible next steps that others might build upon the current work. | ☑ | ☐ | ☐ | 12-14 |
|  | 52 | Strengths and limitations (e.g., bias and generalizability issues)  Clearly state the strengths and the limitations of the current work. Any issue that may lead to potential bias, uncertainty, reproducibility, robustness, and generalizability problems should be declared. | ☑ | ☐ | ☐ | 12-14 |
| Open Science |  |  |  |  |  |  |
| *Data availability* | 53 | Sharing images along with segmentation data  [Please note this item is “not essential” but “recommended”] Provide relevant raw or processed image data considering the regulatory constraints of the institutions involved. Segmentation data can also be shared unless the segmentation is done as part of the workflow. In situations where sharing of the entire dataset is not possible, an end-to-end analysis workflow applied to a representative sample, or a public dataset with similar characteristics can facilitate the ability of the readers in reproducing key components of the analysis [39]. Also, specify the reason if the data is not available. | ☐ | ☑ | ☐ |  |
|  | 54 | Sharing radiomic feature data  Share selected radiomic feature data along with clinical variables or labels with the public, if possible (i.e., in accordance with the regulatory constraints of the institute). Specify the reason if the radiomic feature data is not available. | ☐ | ☑ | ☐ |  |
| *Code availability* | 55 | Sharing pre-processing scripts or settings  Share the pre-processing and feature extraction parameter scripts or settings (e.g., YAML file in PyRadiomics or complete textual description). If it is not available in a script format, then the parameter configuration as appeared in the software program can be shared as a screenshot. | ☐ | ☑ | ☐ |  |
|  | 56 | Sharing source code for modeling  Share the modeling scripts [40]. Code scripts should include sufficient information to replicate the presented analysis (e.g., to train and test pipeline), with all dependencies and relevant comments to easily understand and build upon the method. Even if the actual input dataset used cannot be shared, in situations where a similar dataset is available publicly, it should be used to share an example workflow with all pre- and post-processing steps included. Specify the reason in case the source code is not available. | ☐ | ☑ | ☐ |  |
| *Model availability* | 57 | Sharing final model files  Share the final model files for internal or external testing [40]. Describe how inputs should be prepared to use the model. Also, include the source code that was used for pre-processing the input data. Specify the reason in case the final model data is not available. | ☐ | ☑ | ☐ |  |
|  | 58 | Sharing a ready-to-use system  [Please note this item is “not essential” but “recommended”] An easy-to-use tool (e.g., standalone executable applications, notebooks, websites, virtual machines, etc.) can be created and shared with or without source code that is based on the model created [40]. The main aim is to be able to test or validate the model by other research groups. With this approach, users even without experience in machine learning or coding can also test the proposed models. | ☐ | ☑ | ☐ |  |

**Yes**, details provided; **No**, details not provided; **n/a**, not applicable

1. **Survival and resection margin status between cohorts based on PDAC resectability criteria.**

**Supplementary Figure 3**

In summary, we found that breaking up our NAT-treated patients by their pre-operative resectability status (resectable, borderline resectable, and locally advanced), as previously described in the original manuscript (**Table 1**), produced clinical useful prognostic (i.e., OS, DFS) and predictive (i.e., margin status) information, especially in the resectable and borderline patient populations. In detail, we in fact found we could effectively predict DFS (p < 0.01, log-rank test) in the resectable subgroup of our discovery cohort and observed a p value equal to 0.07 (log-rank test) in borderline resectable patients. The same scenario was seen for OS in resectable patients (validation cohort) where the p value again approached statistical significance (p = 0.07, log-rank test) **(Supplementary Figure 3** and **Supplementary Figure 4**). Given the relative low number of patients in each subgroup for each cohort (resectable= 32, borderline resectable=17, and locally advanced=8 for the discovery cohort and resectable= 13, borderline resectable=12, and locally advanced=6 for the validation cohort), reaching full statistical significance or approaching (i.e., 0.07) it in the resectable and borderline patient populations as described above is a noteworthy result and will pave the way for future clinical trials to validate our combined clinical-radiomic based model (BL-DRF-PC) in a prospective fashion. Finally, for RMS (**Supplementary Table 9**) the breakdown in subgroups also yielded interesting results. In the discovery cohort, the AUC for the resectable, borderline, and locally advanced groups, was 0.71, 0.82, and 0.68, respectively. This data needs to be compared to 0.73 which was obtained as an overall performance of our model considering all patients (please see **Figure 4A** and **4B**). Once again breaking up by resectability status showed (potential) clinically useful results similar to the ones obtained for assessing patient prognosis (please see the paragraph above). Moreover, by looking at the model performance in the validation cohort we found an even increased performance of our prediction model in the resectable patient population (0.72 vs. 0.69: resectable vs. all patients).


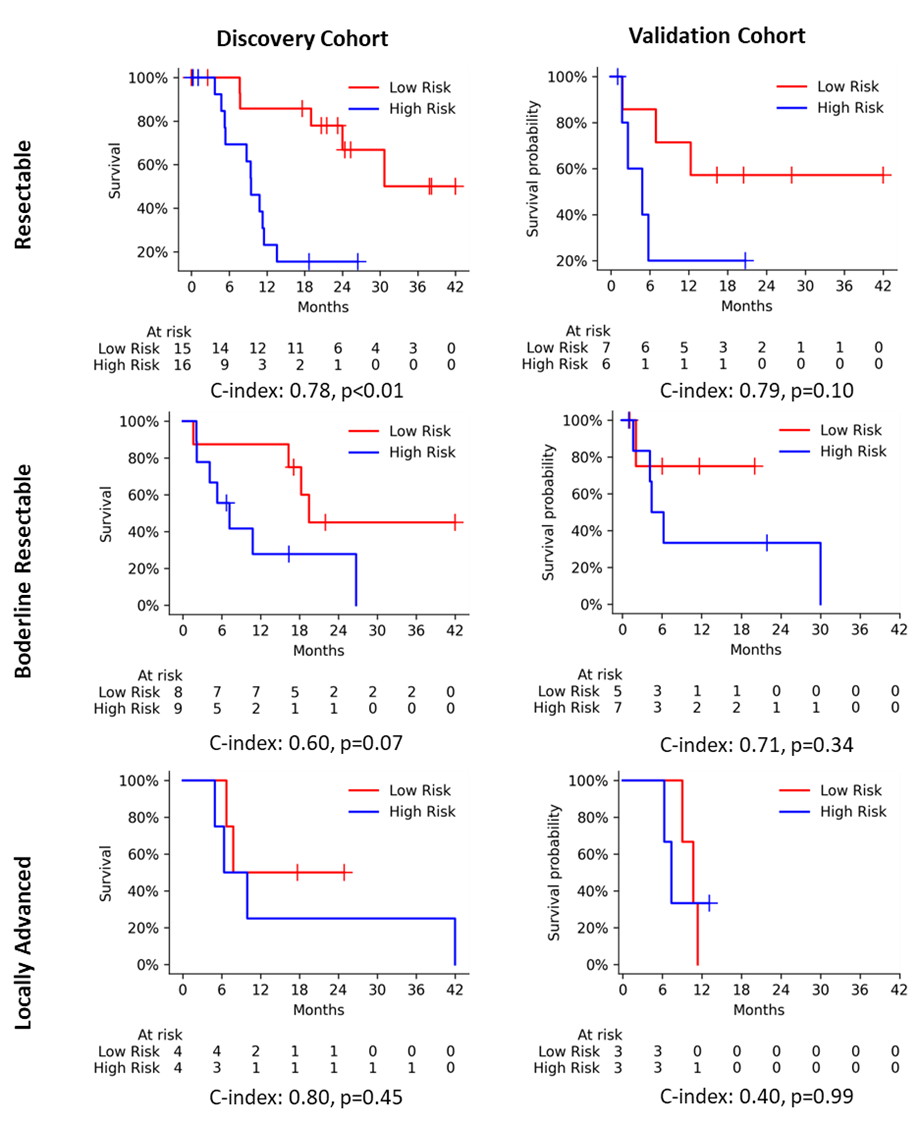


**Supplementary Figure 3**: Disease-free survival (DFS) prediction performance evaluated in C-index and Kaplan-Meier analysis for patient sub-groups. p-values here are from the log-rank tests between the identified high- and low-risk groups.


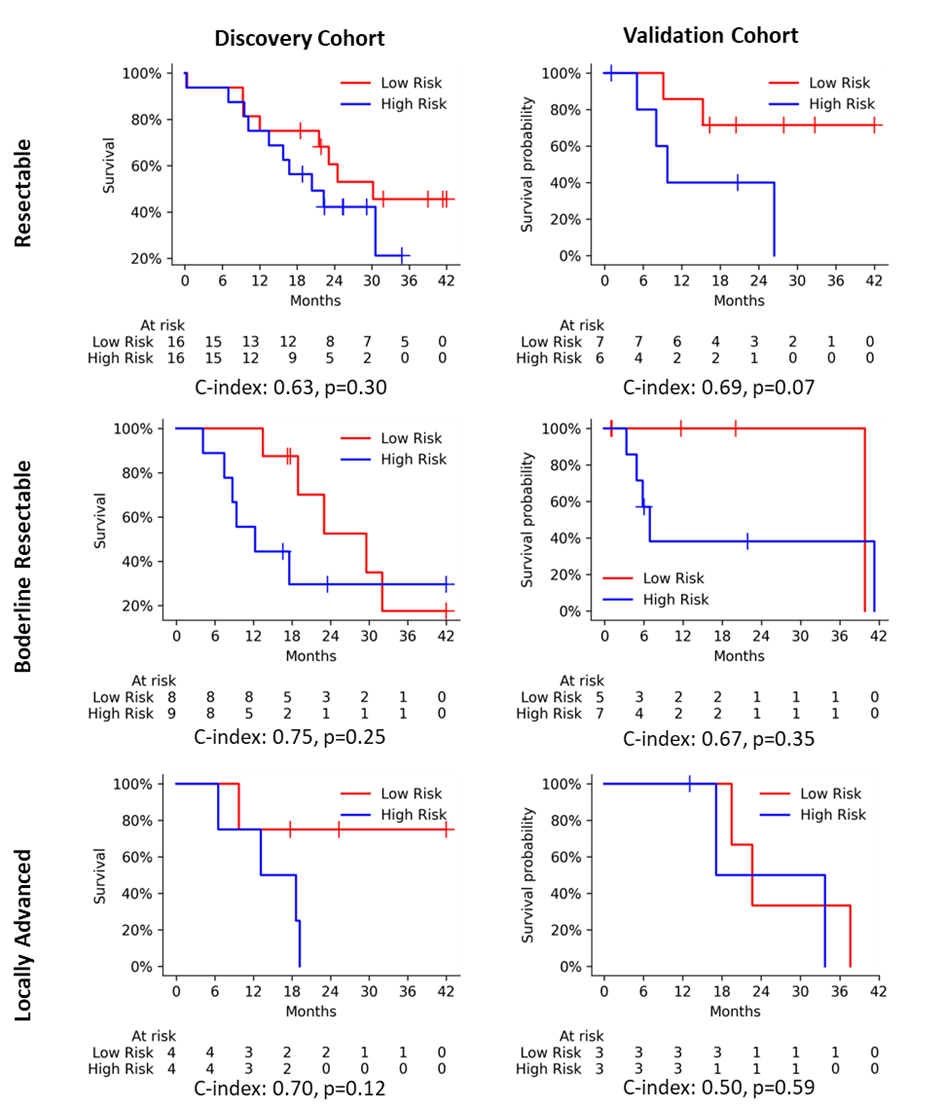


**Supplementary Figure 4**: Overall survival (OS) prediction performance evaluated in C-index and Kaplan-Meier analysis for patient sub-groups. p-values here are from the log-rank tests between the identified high- and low-risk groups.

**Supplementary Table 9**: Resection margin status prediction performance for patient subgroups (resectability status) evaluated by AUC.

|  | Resectable | Borderline Resectable | Locally Advanced | All patients (previously submitted results) |
| --- | --- | --- | --- | --- |
| Discovery Cohort | 0.71 | 0.82 | 0.68 | 0.73 |
| Validation Cohort | 0.72 | 0.66 | 0.42 | 0.69 |

**Reference**

1. Vallieres M, Kay-Rivest E, Perrin LJ, et al. Radiomics strategies for risk assessment of tumour failure in head-and-neck cancer. *Sci Rep-Uk*. Aug 31 2017;7doi:ARTN 10117 10.1038/s41598-017-10371-5

2. Zwanenburg A, Vallières M, Abdalah MA, et al. The image biomarker standardization initiative: standardized quantitative radiomics for high-throughput image-based phenotyping. *Radiology*. 2020;295(2):328-338.

3. Zhou ZG, Folkert M, Iyengar P, et al. Multi-objective radiomics model for predicting distant failure in lung SBRT. *Phys Med Biol*. Jun 7 2017;62(11):4460-4478. doi:10.1088/1361-6560/aa6ae5

4. Wang K, Zhou Z, Wang R, et al. A multi‐objective radiomics model for the prediction of locoregional recurrence in head and neck squamous cell cancers. *Medical Physics*.

5. Zhou Z, Li S, Qin G, Folkert M, Jiang S, Wang J. Multi-objective based radiomic feature selection for lesion malignancy classification. *IEEE J Biomed Health Inform*. Feb 28 2019; doi:10.1109/JBHI.2019.2902298

6. Morgan HE, Wang K, Dohopolski M, et al. Exploratory ensemble interpretable model for predicting local failure in head and neck cancer: the additive benefit of CT and intra-treatment cone-beam computed tomography features. *Quantitative Imaging in Medicine and Surgery*. 2021;

7. Chen X, Zhou M, Wang Z, Lu S, Chang S, Zhou Z. Immunotherapy treatment outcome prediction in metastatic melanoma through an automated multi-objective delta-radiomics model. *Computers in Biology and Medicine*. 2021;138:104916.

8. Rizzo S, Botta F, Raimondi S, et al. Radiomics: the facts and the challenges of image analysis. *Eur Radiol Exp*. Nov 14 2018;2(1):36. doi:10.1186/s41747-018-0068-z

9. Song J, Yin Y, Wang H, Chang Z, Liu Z, Cui L. A review of original articles published in the emerging field of radiomics. *Eur J Radiol*. Jun 2020;127:108991. doi:10.1016/j.ejrad.2020.108991

10. Chen Y, Chen TW, Wu CQ, et al. Radiomics model of contrast-enhanced computed tomography for predicting the recurrence of acute pancreatitis. *Eur Radiol*. Aug 2019;29(8):4408-4417. doi:10.1007/s00330-018-5824-1

11. Dalal V, Carmicheal J, Dhaliwal A, Jain M, Kaur S, Batra SK. Radiomics in stratification of pancreatic cystic lesions: Machine learning in action. *Cancer Lett*. Jan 28 2020;469:228-237. doi:10.1016/j.canlet.2019.10.023

12. Healy GM, Salinas-Miranda E, Jain R, et al. Pre-operative radiomics model for prognostication in resectable pancreatic adenocarcinoma with external validation. *European Radiology*. 2021:1-14.

13. Janssen BV, Verhoef S, Wesdorp NJ, et al. Imaging-based Machine-learning Models to Predict Clinical Outcomes and Identify Biomarkers in Pancreatic Cancer: A Scoping Review. *Annals of surgery*. 2021;
